# Gene-specific DNA methylation profiles and LINE-1 hypomethylation are associated with myocardial infarction risk

**DOI:** 10.1186/s13148-015-0164-3

**Published:** 2015-12-24

**Authors:** Simonetta Guarrera, Giovanni Fiorito, N. Charlotte Onland-Moret, Alessia Russo, Claudia Agnoli, Alessandra Allione, Cornelia Di Gaetano, Amalia Mattiello, Fulvio Ricceri, Paolo Chiodini, Silvia Polidoro, Graziella Frasca, Monique W. M. Verschuren, Jolanda M. A. Boer, Licia Iacoviello, Yvonne T. van der Schouw, Rosario Tumino, Paolo Vineis, Vittorio Krogh, Salvatore Panico, Carlotta Sacerdote, Giuseppe Matullo

**Affiliations:** Human Genetics Foundation (HuGeF), Via Nizza 52, Turin, I-10126 Torino Italy; Medical Sciences Department, University of Turin, Turin, Italy; Julius Center for Health Sciences and Primary Care, UMC Utrecht, Utrecht, The Netherlands; Epidemiology and Prevention Unit, Fondazione IRCCS Istituto Nazionale Tumori, Milan, Italy; Department of Clinical and Experimental Medicine, Federico II University, Naples, Italy; Cancer Epidemiology, CPO Piemonte, Turin, Italy; Department of Public Health, Second University of Naples, Naples, Italy; Cancer Registry and Histopathology Unit, “Civile—M.P. Arezzo” Hospital, ASP 7, Ragusa, Italy; Centre for Nutrition, Prevention and Health Services, National Institute for Public Health and the Environment, Bilthoven, The Netherlands; Department of Epidemiology and Prevention, IRCCS Istituto Neurologico Mediterraneo Neuromed, Pozzilli, IS Italy; Epidemiology and Public Health, Imperial College London, London, UK

**Keywords:** DNA methylation, Myocardial infarction, Early biomarkers, Association study, Risk prediction, Risk stratification

## Abstract

**Background:**

DNA methylation profiles are responsive to environmental stimuli and metabolic shifts. This makes DNA methylation a potential biomarker of environmental-related and lifestyle-driven diseases of adulthood. Therefore, we investigated if white blood cells’ (WBCs) DNA methylation profiles are associated with myocardial infarction (MI) occurrence.

Whole-genome DNA methylation was investigated by microarray analysis in 292 MI cases and 292 matched controls from the large prospective Italian European Prospective Investigation into Cancer and Nutrition (EPIC) cohort (EPICOR study). Significant signals (false discovery rate (FDR) adjusted *P* < 0.05) were replicated by mass spectrometry in 317 MI cases and 262 controls from the Dutch EPIC cohort (EPIC-NL). Long interspersed nuclear element-1 (LINE-1) methylation profiles were also evaluated in both groups.

**Results:**

A differentially methylated region (DMR) within the zinc finger and BTB domain-containing protein 12 (*ZBTB12*) gene body and LINE-1 hypomethylation were identified in EPICOR MI cases and replicated in the EPIC-NL sample (*ZBTB12*-DMR meta-analysis: effect size ± se = −0.016 ± 0.003, 95 % CI = −0.021;−0.011, *P* = 7.54 × 10^−10^; LINE-1 methylation meta-analysis: effect size ± se = −0.161 ± 0.040, 95 % CI = −0.239;−0.082, *P* = 6.01 × 10^−5^).

Moreover, cases with shorter time to disease had more pronounced *ZBTB12*-DMR hypomethylation (meta-analysis, men: effect size ± se = −0.0059 ± 0.0017, *P*_TREND_ = 5.0 × 10^−4^; women: effect size ± se = −0.0053 ± 0.0019, *P*_TREND_ = 6.5 × 10^−3^) and LINE-1 hypomethylation (meta-analysis, men: effect size ± se = −0.0010 ± 0.0003, *P*_TREND_ = 1.6 × 10^−3^; women: effect size ± se = −0.0008 ± 0.0004, *P*_TREND_ = 0.026) than MI cases with longer time to disease.

In the EPIC-NL replication panel, DNA methylation profiles improved case-control discrimination and reclassification when compared with traditional MI risk factors only (net reclassification improvement (95 % CI) between 0.23 (0.02–0.43), *P* = 0.034, and 0.89 (0.64–1.14), *P* < 1 × 10^−5^).

**Conclusions:**

Our data suggest that specific methylation profiles can be detected in WBCs, in a preclinical condition, several years before the occurrence of MI, providing an independent signature of cardiovascular risk. We showed that prediction accuracy can be improved when DNA methylation is taken into account together with traditional MI risk factors, although further confirmation on a larger sample is warranted. Our findings support the potential use of DNA methylation patterns in peripheral blood white cells as promising early biomarkers of MI.

**Electronic supplementary material:**

The online version of this article (doi:10.1186/s13148-015-0164-3) contains supplementary material, which is available to authorized users.

## Background

Cardiovascular diseases (CVDs) are a leading cause of mortality, morbidity, and hospitalization in the adult population in Western countries and a major challenge for developing countries that follow a Westernized-lifestyle. Great attention has been given so far to lifestyle-related CVD risk factors, such as unhealthy diet, smoking habits, and lack of physical activity, whose deleterious effects may be prevented through major lifestyle changes or medical treatments. Apart from monogenic disorders associated with cardiovascular risk (e.g., hypertrophic cardiomyopathy, familial hypercholesterolemia), there is a strong evidence that a family history of CVD and stroke enhances individual CVD risks in relatives as compared with a general population that points out the importance of genetic factors in the etiology of CVDs.

Recent genome-wide association studies (GWASs) reported several potential genetic risk factors for CVDs or intermediate disease phenotypes such as type 2 diabetes, obesity and overweight [1], hypertension [2], and altered lipid profiles [3], underlying the importance of the genetic component. However, the contribution of common genetic variants to non-monogenic CVDs is likely to act in combination with environmental factors or via epistatic (gene-gene or gene-environment) interactions. As gene-environment interactions are thought to be mediated by epigenetic modifications of the genome, epigenetic regulation can be rewarded as the boundary between the inherited genomic asset and the environment, potentially playing a major role in disease onset and severity [4]: epigenetic changes are in fact dynamic, can be modified both during the early in utero development stages and across lifetime by environmental factors as well as diseases, and may be reversible reflecting environmental changes [5, 6]. DNA methylation at CpG dinucleotides is an epigenetic mechanism mainly involved in gene expression regulation. DNA methylation patterns across the genome are not uniform: genetic regions spanning gene locations have variable DNA methylation profiles which are linked to regulatory functions (e.g., gene promoter methylation/demethylation regulates gene expression) and structural functions in shaping local chromatin structures [7, 8]; instead, intergenic regions are usually heavily methylated, since about 45 % of the mammalian genome consists of transposable and viral elements that are silenced by methylation [9]. Methylation levels of the repetitive long interspersed nuclear element-1 (LINE-1) are generally considered as a proxy for global DNA methylation, as LINE-1 elements are widely distributed in the genome and usually heavily methylated in the majority of normal tissues. LINE-1 hypomethylation has previously been associated with ischemic heart disease and stroke [10] and with altered levels of LDL and HDL [11].

Altered DNA methylation profiles have been linked to oxidative stress [12], atherosclerosis [13], ageing [14, 15], and a variety of human diseases ranging from neurological and autoimmune disorders to cancer [16–18]. In addition to individual constitutive DNA methylation profiles that could per se be associated with cardiovascular outcomes [19], subtle and progressive DNA methylation alterations mediated by lifestyle and environmental exposures may in fact lead to dysregulation of several metabolic pathways during lifetime and ultimately to cardiovascular damage and disease [20]. However, the few reports linking cardiovascular outcomes to DNA methylation measured in blood cells or vascular tissue [21–23] did not provide conclusive evidences of DNA methylation involvement in CVD.

Apart from few reports of single CpG associations with a disease or a phenotype, it is usually the cumulative methylation profile of neighboring CpG sites to be more likely associated to a potential functional effect of the methylation status, and the search for differentially methylated regions (DMRs) able to differentiate groups of subjects with different phenotypes or outcomes of interest is a common approach. Along this line, we conducted an epigenome-wide association study (EWAS) to identify DMRs and LINE-1 methylation profiles associated to myocardial infarction (MI) risk in the cardiovascular section (EPICOR) of the Italian cohort of the European Prospective Investigation into Cancer and Nutrition (EPIC) study and replicated statistically significant findings in an independent case-control study nested in the Dutch EPIC cohort (EPIC-NL) with comparable biological samples and information.

Furthermore, we tested whether MI risk prediction accuracy can be improved when DNA methylation profiles, measured at baseline in a preclinical condition, are taken into account together with traditional MI risk factors.

## Results

Descriptive statistics of the sample are reported in Table 1; details on EPIC cohorts are provided in Additional file 1. Statistically significant differences between cases and controls were found in smoking habits, body mass index (BMI) and/or waist-to-hip ratio (WHR), serum lipid profile, and blood pressure, in both the discovery (EPICOR) and the replication (EPIC-NL) studies (Table 1).

**Table 1:** EPICOR and EPIC-NL sample descriptive

	EPICOR men				EPICOR women				EPIC-NL men		EPIC-NL women			
	Cases	Controls			Cases	Controls			Cases	Controls	Cases	Controls		
	(*N* = 188)	(*N* = 188)			(*N* = 104)	(*N* = 104)			(*N* = 116)	(*N* = 83)	(*N* = 201)	(*N* = 179)		
	*n* (%)	*n* (%)			*n* (%)	*n* (%)			*n* (%)	*n* (%)	*n* (%)	*n* (%)		
Center														
Varese	42 (22.34)	42 (22.34)			67 (65.69)	67 (65.69)								
Ragusa	19 (10.11)	19 (10.11)			3 (2.94)	3 (2.94)								
Turin	127 (67.55)	127 (67.55)			23 (22.55)	23 (22.55)								
Naples	–	–			11 (10.78)	11 (10.78)								
Utrecht									–	–	149 (74.13)	140 (78.21)		
Bilthoven									116 (100)	83 (100)	52 (25.87)	39 (21.79)		
Smoking status														
Never	33 (17.55)	50 (26.60)			53 (50.96)	73 (70.19)			19 (16.38)	18 (21.69)	60 (29.85)	85 (47.49)		
Former	70 (37.23)	87 (46.28)		*	11 (10.58)	13 (12.50)		*	34 (29.31)	32 (38.55)	48 (23.88)	48 (26.81)		*
Current	85 (45.21)	51 (27.13)			40 (38.46)	18 (17.31)			62 (53.45)	33 (39.76)	89 (44.28)	45 (25.14)		
NA									1 (0.86)		4 (1.99)	1 (0.56)		
Menopausal Status														
Premenopause					27 (25.96)	26 (25.00)					43 (21.39)	42 (23.46)		
Post-menopause					77 (74.04)	78 (75.00)					158 (78.61)	137 (76.54)		
	Mean ± sd	Mean ± sd			Mean ± sd	Mean ± sd			Mean ± sd	Mean ± sd	Mean ± sd	Mean ± sd		
Age at recruitment (years)	50.98 ± 6.93	50.92 ± 7.01			55.02 ± 7.40	55.01 ± 7.51			51.51 ± 7.68	51.11 ± 8.30	58.56 ± 8.74	59.30 ± 8.12		
Avg. follow-up (years)	12.98 ± 2.29	13.26 ± 2.06			12.24 ± 1.97	12.66 ± 1.16			13.07 ± 5.21	15.25 ± 2.31**	11.61 ± 4.92	14.27 ± 2.62**		
Avg. TTD (years)	7.14 ± 3.88	–			6.54 ± 3.57	–			5.44 ± 3.22	–	5.76 ± 3.06	–		
BMI (kg/m2)	27.05 ± 2.96	26.35 ± 3.12**			26.95 ± 4.69	25.93 ± 5.11			27.12 ± 3.30	27.08 ± 3.15	26.47 ± 4.24	26.05 ± 4.17		
WHR	0.94 ± 0.06	0.93 ± 0.06**			0.83 ± 0.06	0.79 ± 0.06**			0.95 ± 0.08	0.94 ± 0.08	0.82 ± 0.07	0.80 ± 0.07**		
Total cholesterol (mmol/L)	6.10 ± 1.12	5.86 ± 1.22			6.42 ± 1.23	6.36 ± 1.16			6.24 ± 0.95	5.79 ± 0.97**	5.51 ± 0.98	5.27 ± 0.98**		
LDL cholesterol (mmol/L)	3.94 ± 1.00	3.60 ± 1.01**			4.07 ± 1.15	3.97 ± 1.01			3.60 ± 0.94	3.34 ± 0.92	3.44 ± 0.82	3.19 ± 0.77**		
HDL cholesterol (mmol/L)	1.30 ± 0.29	1.48 ± 0.37**			1.55 ± 0.39	1.76 ± 0.41**			1.11 ± 0.28	1.12 ± 0.28	1.16 ± 0.33	1.27 ± 0.36**		
Triglycerides (mmol/L)	1.89 ± 0.99	1.71 ± 1.04			1.74 ± 1.43	1.38 ± 0.57**			2.28 ± 1.27	2.28 ± 1.46	1.77 ± 0.98	1.54 ± 0.91**		
SBP (mmHg)	137.47 ± 16.56	135.04 ± 19.03			144.36 ± 9.77	136.76 ± 10.08**			134.43 ± 17.61	128.51 ± 14.93**	138.50 ± 22.23	133.59 ± 20.76**		
DBP (mmHg)	85.38 ± 8.96	84.97 ± 10.80			86.08 ± 22.13	84.97 ± 19.02			84.96 ± 10.71	80.60 ± 10.19**	81.99 ± 11.20	79.22 ± 11.18**		
Alcohol (gr/day)	23.46 ± 20.26	24.98 ± 20.90			6.29 ± 10.78	8.76 ± 15.43			19.42 ± 21.89	18.41 ± 22.27	7.55 ± 11.24	8.44 ± 12.21		

*LDL* low-density lipoprotein, *HDL* high-density lipoprotein, *SBP* systolic blood pressure, *DBP* diastolic blood pressure, *TTD* time to disease, *WHR* waist-to-hip ratio

*Chi-square test *P* < 0.05; **t test *P* < 0.05

After raw methylation data quality controls (QCs), and removal of cross-hybridizing and single-nucleotide polymorphism-containing probes, 425,498 CpGs were included into the following analyses.

### Case-control differential methylation

In the EPICOR sample, 25,376 regions with correlated methylation levels were identified with the A-clustering algorithm [24] and subsequently tested for differential methylation between cases and controls (see the “Methods” section): the top-ranking 6 DMRs are reported in Table S1 (Additional file 2). However, only the first region reached statistical significance (false discovery rate (FDR) *Q* < 0.05), i.e., a 15-CpGs cluster within the gene body (exon 1) of the zinc finger and BTB domain-containing protein 12 gene (*ZBTB12*, gene ID: 221527) that was hypomethylated in cases as compared to controls (effect size ± se = −0.019 ± 0.004, 95 % CI −0.03;−0.01, *P* = 1.94 × 10^−7^, *Q* = 0.005). To check for sex-specific effects of the *ZBTB12*-DMR, we stratified EPICOR subjects by sex and found the 15-CpGs cluster still significantly hypomethylated in male cases (effect size ± se = −0.023 ± 0.005, 95 % CI −0.03;−0.01, *P* = 1.06 × 10^−6^), but not in females (effect size ± se = −0.006 ± 0.006, 95 % CI −0.02;0.005, *P* = 0.29). Details on single CpGs are reported in Table S2A (Additional file 2).

The genomic inflation factor for the overall EPICOR sample was lambda = 1.023 (men, lambda = 1.043; women, lambda = 1.017; *Q*-*Q* plots in Additional file 3: Figures S1–S3).

LINE-1 differential methylation was also tested in the EPICOR overall sample by logistic regression analysis: MI cases had statistically significant LINE-1 hypomethylation as compared to controls (effect size ± se = −0.511 ± 0.147, 95 % CI −0.80;−0.22, *P* = 5.00 × 10^−4^). At a sex-stratified analysis, LINE-1 hypomethylation was still statistically significant in men (effect size ± se = −0.520 ± 0.179, 95 % CI −0.87;−0.17, *P* = 0.004), but not in women (effect size ± se = −0.496 ± 0.319, 95 % CI −1.12;−0.13, *P* = 0.12).

Additionally, for *ZBTB12*-DMR, we found a significant sex-methylation interaction (*P* = 0.01), while for LINE-1 we found no evidence of interaction.

Results were replicated on the EPIC-NL panel, where the methylation profile of the same *ZBTB12*-DMR identified in the discovery phase proved consistent with that of the EPICOR discovery sample, with a cluster of 22 contiguous CpGs significantly hypomethylated in Dutch MI cases as compared to controls (effect size ± se = −0.013 ± 0.004, 95 % CI −0.02;−0.005 *P* = 5.82 × 10^−4^). Details on *ZBTB12*-DMR single CpGs for the EPIC-NL study are reported in Table S2B (Additional file 2).

At a sex-stratified analysis, *ZBTB12*-DMR was hypomethylated in both EPIC-NL men (effect size ± se = −0.014 ± 0.007, 95 % CI −0.03;−0.001, *P* = 0.034) and women (effect size ± se = −0.012 ± 0.004, 95 % CI −0.02;−0.004, *P* = 0.006), with effect sizes more comparable between men and women than in the EPICOR sample.

In the EPIC-NL panel, LINE-1 mean methylation levels were lower than those of EPICOR, with an average methylation of about 0.8 in EPICOR subjects (men: mean ± sd = 0.844 ± 0.007; women: mean ± sd = 0.843 ± 0.007) and about 0.6 in EPIC-NL subjects (men: mean ± sd = 0.624 ± 0.029; women: mean ± sd = 0.613 ± 0.023). As seen in the EPICOR panel, we found LINE-1 hypomethylation also in Dutch cases as compared to controls, although with a milder effect (effect size ± se = −0.132 ± 0.042, 95 % CI −0.21;−0.05, *P* = 0.001). In EPIC-NL men, the sex-stratified LINE-1 analysis showed an effect size similar to that found in EPICOR (effect size ± se = −0.40 ± 0.085, 95 % CI −0.57;−0.23, *P* = 2.22 × 10^−6^), while in EPIC-NL women the effect was much lower and statistically non-significant (effect size ± se = −0.016 ± 0.046, 95 % CI −0.11;0.07, *P* = 0.73).

In the Dutch panel, we found no evidence of sex-methylation interaction for *ZBTB12*-DMR, while we found a statistically significant interaction for LINE-1 (*P* = 0.0003).

The observation of sex-methylation interactions in both the discovery and replica panels, and further considerations addressed in the “Discussion” section, suggested to consider men and women separately in all the subsequent analyses.

To achieve an overall estimate of the effects of *ZBTB12*-DMR and LINE-1 methylation across the two subject panels, we performed a meta-analysis of the EPICOR and EPIC-NL studies.

The estimated *ZBTB12*-DMR effects were effect size ± se = −0.016 ± 0.003 in the overall sample (*P* = 7.54 × 10^−10^, 95 % CI = −0.021;−0.011, Cochran’s *Q* = 0.005, d.f. = 1, *P*_HET_ = 0.83), effect size ± se = −0.020 ± 0.004 in men (*P* = 1.82 × 10^−7^, 95 % CI = −0.027;−0.012, Cochran’s *Q* = 0.007, d.f. = 1, *P*_HET_ = 0.79), and effect size ± se = −0.010 ± 0.003 in women (*P* = 0.005, 95 % CI = −0.017;−0.003, Cochran’s *Q* = 0.004, d.f. = 1, *P*_HET_ = 0.84).

The estimated LINE-1 effects were effect size ± se = −0.161 ± 0.040 in the overall sample (*P* = 6.01 × 10^−5^, 95 % CI = −0.239;−0.082, Cochran’s *Q* = 0.85, d.f. = 1, *P*_HET_ = 0.35), effect size ± se = −0.422 ± 0.076 in men (*P* = 3.42 × 10^−8^, 95 % CI = −0.572;−0.272, Cochran’s *Q* = 0.06, d.f. = 1, *P*_HET_ = 0.81), and effect size ± se = −0.025 ± 0.046 in women (*P* = 0.576, 95 % CI = −0.115;0.064, Cochran’s *Q* = 0.70, d.f. = 1, *P*_HET_ = 0.40).

### DNA methylation and MI risk

The MI risk associated to *ZBTB12*-DMR and LINE-1 hypomethylation was estimated in the EPIC-NL replica panel: recursively partitioned mixture model (RPMM) classes and LINE-1 class (as defined in the “Methods” section) were tested for association with MI under different models, from unadjusted to fully adjusted.

When comparing the *ZBTB12*-DMR lowest methylation class (RPMM3) with the highest methylation class (RPMM0), we found MI risk to be significantly associated with hypomethylation in the EPIC-NL women (fully adjusted, OR = 2.75, 95 % CI 1.39–5.45, *P* = 0.004), while in EPIC-NL men the association was statistically non-significant (fully adjusted, OR = 2.60, 95 % CI 0.79–8.56, *P* = 0.116), although direction and effect size were similar.

We also found a higher MI risk associated with LINE-1 lower methylation class in EPIC-NL men (fully adjusted, OR = 1.95, 95 % CI 1.02–3.71, *P* = 0.043, ref. group above the median). No difference was found in EPIC-NL women (fully adjusted, OR = 1.05, 95 % CI 0.65–1.67, *P* = 0.850) (Additional file 2: Table S3A).

The same analysis was performed on the EPICOR discovery sample: even though in this case the ORs cannot be considered as indicative of a true estimate of risk being EPICOR subjects the discovery panel, the analysis was nevertheless done to assess whether the progressive inclusion in the model of additional variables, namely traditional risk factors (TRFs), could modify the estimate of risk or, on the contrary, if DNA methylation may independently contribute to MI risk. No significant evidence of inflation/deflation of the DNA methylation-related MI risk estimate was found nor for the EPIC-NL panel nor for the EPICOR panel when progressively adding TRFs as covariates in the model (Additional file 2: Tables S3A and B).

### Discrimination, reclassification, and calibration on EPIC-NL samples

**Fig. 1:**
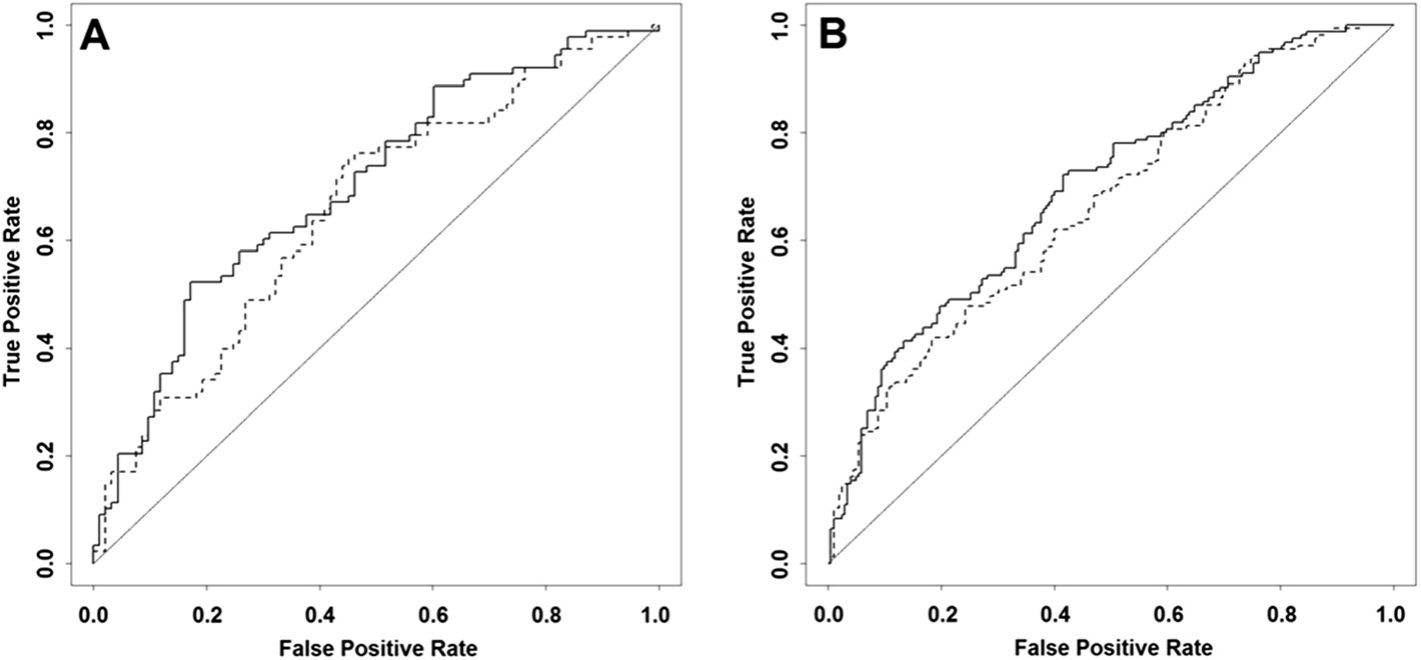
Receiver operating curves (ROC), EPIC-NL validation sample. Model 1 (TRFs, *dotted line*) includes age, sex, center of recruitment, smoking habits, BMI, WHR, lipid levels, blood pressure, menopausal status in women. Model 2 (TRFs + Meth, *solid line*), as model 1 plus *ZBTB12-*RPMM classes, LINE-1 methylation profile. **a** EPIC-NL men. **b** EPIC-NL women. Statistics in Table 2

We assumed two models, including the following: (1) TRFs only and (2) TRFs plus the *ZBTB12*-based RPMM classes and LINE-1 methylation class. According to the net reclassification improvement (NRI) and integrated discrimination improvement (IDI) indices (Table 2), a statistically significant improvement in prediction performance was achieved when adding the DNA methylation profiles to the set of baseline predictors (i.e., TRFs), both for EPIC-NL male and female groups. Furthermore, we found an improvement in discrimination (Table 2, DeLong’s test) comparing the area under the receiver operating curves (AUC) of the two models (Table 2 and Fig. 1), although it was not statistically significant.

**Table 2:** Discrimination and reclassification indices, EPIC-NL validation sample

	AUC_TRF_ (95 % CI)	AUC_TRF + M_ (95 % CI)	DeLong’s test *P*	NRI (95 % CI)	*P*_NRI_	IDI (95 % CI)	*P*_IDI_
EPIC-NL men	0.66 (0.58–0.74)	0.70 (0.63–0.78)	0.147	0.47 (0.19–0.76)	0.001	0.04 (0.01–0.08)	0.004
EPIC-NL women	0.66 (0.61–0.72)	0.69 (0.63–0.74)	0.095	0.23 (0.02–0.43)	0.034	0.03 (0.01–0.05)	0.001

**Fig. 2:**
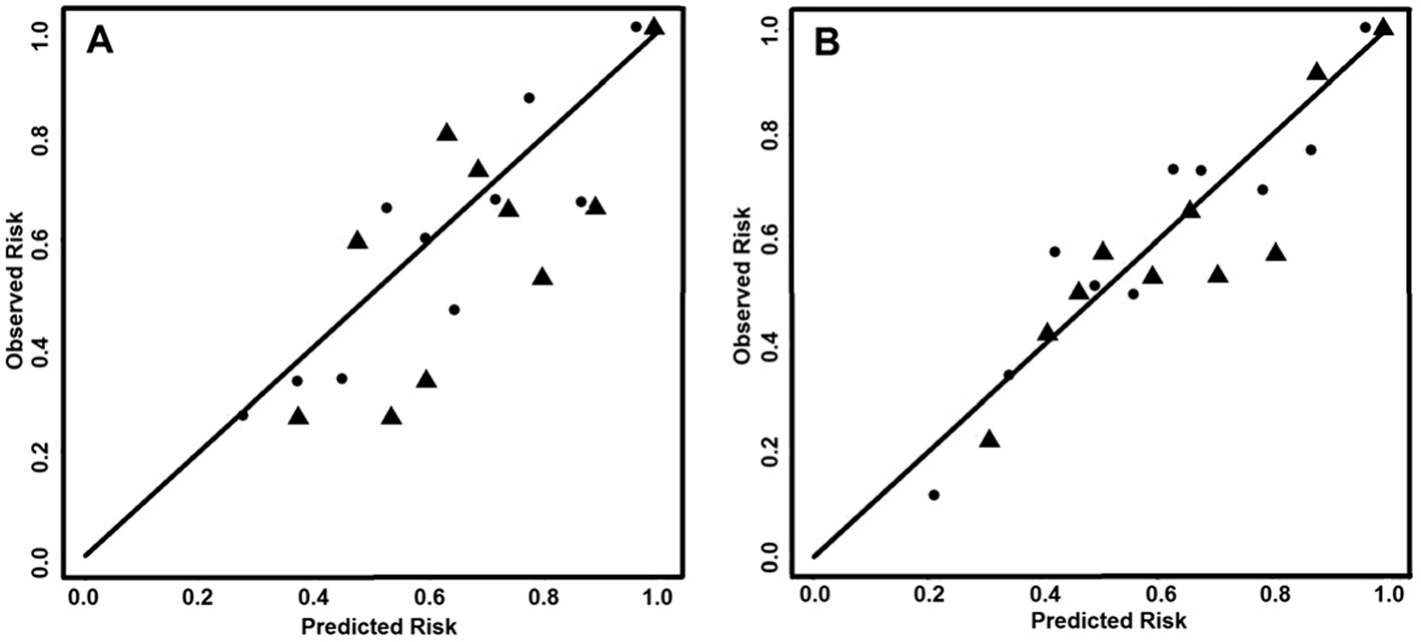
Calibration plots, EPIC-NL validation sample. Goodness of fit, model 1 (TRFs, *triangles*) vs model 2 (TRFs + Meth, *dots*). Hosmer-Lemeshow test: men: *P*_TRF_ = 0.118, *P*_TRF + M_ = 0.414; women: *P*_TRF_ = 0.636, *P*_TRF + M_ = 0.724. **a** EPIC-NL men. **b** EPIC-NL women. Statistics in Table 2

The calibration plots confirmed the goodness of fit of both the TRFs only and TRFs + Methylation models (Fig. 2, Hosmer-Lemeshow test), with a better performance of the second one.

### DNA methylation and time to disease

The trend test on EPICOR and EPIC-NL subjects, stratified by study and by sex, highlighted a more pronounced *ZBTB12*-DMR hypomethylation in cases with shorter time to disease (EPICOR and EPIC-NL meta-analysis, men: *P*_TREND_ = 0.0005, women *P*_TREND_ = 0.0065; Table 3). Similarly, LINE-1 was hypomethylated in cases with shorter time to disease (meta-analysis, men: *P*_TREND_ = 0.0016, women *P*_TREND_ = 0.026; Table 3).

**Table 3:** DNA methylation and time to disease (TTD)

		*ZBTB12*					LINE-1				
TTD class^a^	Range^b^	Effect size	95 % CI	se	*P*_TREND_	Cochran’s *Q*	Effect size	95 % CI	se	*P*_TREND_	Cochran’s *Q*
EPICOR men											
TTD class 1	8.89–14.66										
TTD class 2	5.23–8.88	−0.0054	−0.0090;−0.0018	0.0018	0.0036		−0.0009	−0.0016;−0.0003	0.0003	0.0044	
TTD class 3	0.26–5.20										
EPIC-NL men											
TTD class 1	6.97–12.31										
TTD class 2	3.53–6.86	−0.0093	−0.0182;−0.0005	0.0045	0.0389		−0.0035	−0.0070;0.00003	0.0018	0.0537	
TTD class 3	0.23–3.52										
Meta-analysis		−0.0059	−0.0093;−0.0027	0.0017	0.0005	0.65^c^	−0.0010	−0.0017;−0.0004	0.0003	0.0016	2.03^c^
EPICOR women											
TTD class 1	8.16–14.02										
TTD class 2	4.40–8.06	−0.0056	−0.0106;−0.0007	0.0025	0.0250		−0.0008	−0.0017;0.00004	0.0004	0.0636	
TTD class 3	0.33–4.30										
EPIC-NL women											
TTD class 1	7.35–12.30										
TTD class 2	4.40–7.21	−0.0047	−0.0106;0.0011	0.0030	0.1147		−0.0011	−0.0032;0.0010	0.0011	0.2970	
TTD class 3	0.04–4.38										
Meta-analysis		−0.0053	−0.0091;−0.0015	0.0019	0.0065	0.05^c^	−0.0008	−0.0016;−0.0001	0.0004	0.0263	0.07^c^

^a^Healthy controls (TTD class 0) were used as the reference group. Cases were divided in tertiles (TTD classes 1 to 3)

^b^Minimum and maximum TTD (i.e., time lapse in years from enrollment to occurrence of MI) for each class

^c^d.f. = 1, *P* = not significant

At a post hoc power analysis, our study was well powered (86 and 82 % for male and female groups, respectively) to identify DMRs with effect sizes equal to half of the standard deviation, considering alpha equal to the FDR threshold of significance (*Q* = 0.05).

## Discussion

In this study, we investigated whether white blood cells’ (WBCs) DNA methylation profiles may be associated with MI risk. We examined clusters of adjacent CpG sites with correlated methylation levels under the assumption that they could be more reliable indicators of the underlying biological function than the single CpG methylation measurement. As we found evidences of sex-methylation interactions in both the analyzed panels, in our study the analyses were stratified by sex, in order to account for sex-related differences in DNA methylation profiles of genomic regions, of which “genomic imprinting” is a well-known example, and to account for sex-specific cardiovascular risks. For coronary heart disease, sex differences in incidence, disease manifestations, and mortality are well recognized [25], and men and women seem not to share the same cardiovascular risk factors [26–29]. Moreover, patterns of sex-specific methylation have been reported in literature, and there is a general consensus on the occurrence of sex-biased autosomal DNA methylation in specific genes and regions, although with contrasting results [30–32]. Sex-associated differential DNA methylation in autosomal loci has been reported in genes associated to traits/diseases with different incidence rates according to sex [33], as well as in hormone-related genes, suggesting a differential regulation, potentially exerted via methylation [31]. Differential DNA methylation may account for the differences in metabolic profiles of men and women, possibly leading to the different incidence, prevalence, symptoms, ages at onset, and severity of CVDs reported in literature.

In the EPICOR discovery panel, we identified a 15-CpGs cluster within the *ZBTB12* gene that was significantly differentially methylated in Italian MI cases and controls and that was also significantly hypomethylated in MI cases in the independent Dutch panel. Moreover, *ZBTB12*-DMR showed a trend towards more pronounced hypomethylation in subjects with a short time to disease (TTD) both in the Italian and in the Dutch samples.

*ZBTB12*-DMR spans a ~250-bp region in *ZBTB12* exon 1: although the role of gene-body methylation in transcriptional regulation is not fully understood, yet there are evidences of a role of the first exon’s DNA methylation in transcriptional silencing and, putatively, in alternative splicing [34]. All of our samples belong to the EPIC cohort, for which no biospecimen suitable for transcriptome analyses is available to address the relationship between *ZBTB12* methylation and gene expression levels. To cope with this issue, we explored *ZBTB12* DNA methylation/gene expression relationship in cryopreserved peripheral blood mononuclear cells from ~80 healthy young subjects belonging to another ongoing study, for which we already measured methylation and gene expression levels: in our data, *ZBTB12* messenger RNA (mRNA) abundance was below the background level (as assessed by Illumina HumanHT12 gene expression BeadChip), while *ZBTB12* methylation levels were comparable to that of EPICOR and EPIC-NL controls (data not shown). No relationship was found also with the gene expression levels of the nearby genes (data not shown). Data mining in freely available resources (e.g., BioGPS, AceView, ProteinAtlas, Genome Atlas) confirmed the generalized low *ZBTB12* mRNA level in tissues and cell types, although ZBTB12 protein is detectable in many tissues, including cardiovascular tissues. Although no clear function is described for ZBTB12, this protein is probably involved in transcriptional regulation, like other members of the ZBTB family of methyl-CpG binding proteins (MBPs). This is also supported by its mainly nuclear localization. MBPs bind to methylated DNA and recruit chromatin remodeling co-repressor complexes, resulting in compaction of chromatin into its transcriptionally inactive state [35]. Specifically, members of the ZBTB family function as mediators of epigenetically controlled gene silencing by recognizing symmetrically methylated CpG sites and sequence-specific non-methylated sites [8, 35].

According to the Human Protein Reference Database [36], ZBTB12 (HPRD ID: 15691) directly interacts with Harvey rat sarcoma viral oncogene homolog (HRAS) and Ras-associated protein 1 (RAP1) GTPase-activating protein 1 (RAP1GAP). RAP1GAP downregulates the activity of RAP1, a small GTPase involved in several aspects of cell adhesion, including angiogenesis [37]. HRAS, a member of the RAS oncogene family, is a key transducer in several growth-signaling events that may trigger cardiovascular complications such as angiogenesis and vascular permeability [38] and may be involved in inflammatory proliferative arterial diseases, including atherosclerosis and restenosis after angioplasty [39]. The RAS-MEK-ERK cascade has been described as implicated in cardiac hypertrophy and heart failure, and ERK signal transduction pathways were associated with cardiac hypertrophy [40].

In addition to gene-/region-specific DNA methylation, we investigated the cumulative DNA methylation profile of LINE-1 repetitive sequences and found LINE-1 hypomethylation in MI cases, statistically significant in men in both panels, but not in women. LINE-1 hypomethylation was associated to cardiovascular-related traits in previous studies [10, 11], and it is associated to MI and shorter TTD in the present study. DNA hypomethylation is regarded as a cause of genomic instability, and as a matter of fact, LINE-1 hypomethylation was found in several conditions, including cancer [41], autoimmune diseases [42], and CVDs [10]. Specifically, global hypomethylation of genomic DNA and gene-specific methylation profiles have been associated to conditions already known to predispose to CVDs, such as cellular ageing [43], atherosclerotic plaques [44], menopausal state, and osteoporosis [45]. On the other hand, LINE-1 hypomethylation could simply be a marker of increased WBC proliferation due to inflammatory or immunological responses which are known to be active during cardiovascular pathogenic processes [10]. In vitro experiments on mouse embryonic stem cells showed that folate deficiency affected the homeostasis of folate-mediated one-carbon metabolism, leading to reduced LINE-1 methylation [46]. In a targeted analysis, we recently demonstrated on a subset of the EPICOR cohort (206 MI cases and 206 matched controls) an inverse relationship between B vitamin intake and DNA methylation of genes belonging to one-carbon metabolism and homocysteine pathways [20]. These previous observations, together with our current finding of LINE-1 hypomethylation in cases compared to healthy controls, suggest a link between DNA methylation patterns and CVD risk conferred by low folate and B vitamin intake that is worthy of further investigation.

Overall, this study analyzed 609 cases and 554 controls and was sufficiently powered to detect effects of the magnitude we found. The discovery and the replica panels share homogeneous features: both belong to the European EPIC cohort, subjects were all enrolled in the 1990s, and biosamples were collected and stored at enrollment according to shared standard protocols [47]. Nevertheless, a limitation of the study is that while EPICOR cases and controls were matched by age, sex, center, and season of recruitment, this could not be achieved for the EPIC-NL sample, since a DNA sample suitable for methylation analysis was not available for all the subjects enrolled in the EPIC-NL.

Another limitation is that the assessment of the methylation levels was done with different methods for the two panels. However, our approach that considered the regional methylation profile as a whole instead of single CpGs may contribute to overcome the bias due to measure errors at the single CpG level, as highlighted by the correlation between the methylation measures of 16 control samples assayed with both BeadChip and MassARRAY assays (Additional file 1). Moreover, although the CpG positions assayed with the two methods are not exactly the same due to technical constrains (Additional file 1 and Additional file 3: Figure S4), still the analysis of methylation data collected with each one of the two different techniques highlighted a cluster of CpGs with correlated methylation levels within exon 1 of *ZBTB12*, hypomethylated in MI cases vs controls. This complies with our study design assumption that the methylation status of multiple CpGs with correlated methylation could better describe the cumulative methylation status of the underlying region and that this could be potentially related to the underlying biological function, if any. The same goes for LINE-1 methylation, which is defined as the cumulative DNA methylation status of the several CpGs located in LINE-1 sequences across the genome. Also in this case, different portions of LINE-1 sequence were investigated with the two techniques, i.e., CpGs scattered across the whole LINE-1 sequence were analyzed on the BeadChip, whereas CpGs within base pairs 335–767 of the LINE-1 promoter (GenBank accession number X58075.1) were analyzed by MassARRAY according to Wang et al. [48] (Additional file 3: Figure S4).

Despite slight differences between EPICOR and EPIC-NL panels in LINE-1 average methylation levels, arguably due to the use of different methods and different assayed CpGs, our results highlighted the same effect trend in both the EPIC sub-cohorts.

When included in the same multivariate models, the estimated risks associated to *ZBTB12*-DMR and LINE-1 methylation profiles were not attenuated by the adjustment for known risk factors (Additional file 2: Table S3A and B), suggesting that they independently contribute to MI risk estimate.

Moreover, we observed that discrimination between MI cases and controls and prediction accuracy both improved when DNA methylation was taken into account together with TRFs, suggesting the DNA methylation could be an independent predictor of MI risk, although further confirmation on a larger sample is warranted.

Our results highlight the possibility to identify MI-related methylation marks on DNA from blood samples drawn in a preclinical condition, for some subjects many years before the MI. Unfortunately, due to the initial EPIC study design that envisaged only one blood sampling at enrollment time, it was not possible to monitor individual DNA methylation level changes at different time points. Further replication in additional cohorts with prospective design and biospecimens sampled at multiple points along time is warranted to elucidate DNA methylation changes across time, from a “healthy” status to MI. This will allow a better estimation of the *ZBTB12*-DMR and LINE1 demethylation rates associated with increased MI risk, in the view of a personalized risk assessment that will take into account TRFs and MI risk biomarkers, such as DNA methylation profiles.

## Conclusions

To the best of our knowledge, this is the first paper reporting an association between MI risk and DNA methylation profiles identified from epigenome-wide data in prospectively collected subjects with well-recorded clinical endpoints and replicated in an independent sample form the same large European prospective cohort.

Taken together, the reported results suggest the possible role of DNA methylation patterns in peripheral blood white cells as promising early MI biomarkers to be potentially used, together with TRFs, for individual MI risk assessment.

## Methods

### Study population

For the discovery phase, 292 MI cases and 292 matched healthy controls were recruited among those enrolled in the EPICOR study [49], a case-cohort study nested within the EPIC-Italy prospective cohort (~50,000 participants) [50]. All EPICOR cases developed MI after recruitment (average time to diagnosis 6.90 years). Cases were identified at cohort follow-up from hospital discharge databases and were then matched with healthy controls from the same cohort without evidence of MI at follow-up. Matching parameters were age at recruitment (±1.5 years), sex, center, and season of recruitment. Results from the discovery phase were replicated in an independent sample of 317 Dutch subjects from the prospective EPIC-NL cohort [51] who developed MI during follow-up (average time to diagnosis 5.64 years) and 262 unmatched healthy controls from the same cohort. Details on anthropometrics, lifestyle, biochemical measurements, and MI definition are provided in Additional file 1.

### Ethical considerations

Our study complies with the Declaration of Helsinki principles and conforms to ethical requirements. All volunteers signed an informed consent form at enrollment in the respective studies. The EPIC study protocol was approved by Ethics Committees of the International Agency for Research on Cancer (Lyon, France), as well as by local Ethical Committees of the participant centers. The EPICOR study was approved by the Ethical Committee of the Human Genetics Foundation (Turin, Italy). For the Dutch EPIC samples, approval was obtained by the Institutional Review Board of the University Medical Center Utrecht (Utrecht, the Netherlands) and the Medical Ethical Committee of TNO Nutrition and Food Research (Zeist, the Netherlands).

### DNA methylation measurement

DNA methylation was measured in DNA from WBCs collected at subject enrollment into EPIC and stored in liquid nitrogen [47]. The Infinium HumanMethylation450 BeadChip (Illumina Inc., San Diego, CA, USA) and the MALDI-TOF mass spectrometry methylation assay (Sequenom Inc., San. Diego, CA, USA) were used for the discovery phase and the replication phase, respectively. Laboratory methods for DNA methylation level measurements are detailed in Additional file 1.

### Whole-genome methylation data quality control and normalization procedures

DNA methylation levels were measured as Beta-values, ranging from 0 to 1. We excluded the following from the analyses: (i) single Beta-values with detection *P* value ≥ 0.01; (ii) CpG loci with detection *P* value ≥ 0.01 in more than 20 % of the assayed samples; (iii) probes containing SNPs with MAF ≥ 0.05 in the CEPH (Utah residents with ancestry from Northern and Western Europe, CEU) population; and (iv) samples with a global call rate ≤95 %.

From the 435,457 CpGs that passed QCs (~95 % of BeadChip content), we further removed 9959 CpGs whose methylation signal was detected by cross-hybridizing and SNPs-containing probes [52].

A total of 292 matched case-control pairs and 425,498 CpG sites were used in the following analyses.

Background normalization was performed on raw methylation data according to Marabita et al. [53].

### Statistical analyses

Statistical analyses were conducted using the open source R v3.0.1 package [54].

Analyses were performed stratifying by sex, in order to account for the occurrence of sex-specific DNA methylation and for the different cardiovascular risk profiles between men and women (see the “Discussion” section). Descriptive statistics of sample characteristics, anthropometrics, lipid profiles, hypertension, and lifestyle habits (smoke, alcohol consumption) was performed.

### Case-control DMR analysis

We analyzed the EPICOR methylation data (discovery phase, 425,498 CpGs) with the A-clustering algorithm [24] to identify clusters of two or more neighboring CpGs with correlated methylation levels.

The association between each one of the identified methylation clusters and case-control status was tested by generalized estimating equations (GEE) [55] to identify DMRs between MI cases and controls. We adjusted the analyses for matching variables (age at recruitment, center, season of recruitment, sex in the overall analyses), estimated WBC composition (for the EPICOR panel only), and the major cardiovascular risk factors [56], i.e., smoking status, BMI, blood pressure, and physical activity (for the EPICOR panel only). EPICOR sample analyses were additionally adjusted for “control probes” principal components, while EPIC-NL analyses did not require batch correction (see Additional file 1: Removal of technical biases).

As fasting glucose measurement was missing for >20 % of the EPICOR and EPIC-NL samples, glucose level was excluded from the adjustment covariates. Lipid levels were missing for 48 EPICOR subjects: lipid levels were omitted as covariates too, after verification that inclusion or exclusion of this parameter did not substantially affect the results (Additional file 1).

Due to the small number of subjects with incident diabetes identified at follow-up (*n* = 9), diabetes was not included in the covariate list.

DMRs with FDR *Q* value < 0.05 were considered statistically significant and investigated in the EPIC-NL sample with the same statistical approach. The *Q* statistic [57] was used to assess heterogeneity between the two sample panels: provided no heterogeneity was found, an inverse variance-weighted fixed effect meta-analysis was additionally carried out to achieve an overall estimate of the two studies.

### Case-control LINE-1 methylation analyses

To analyze LINE-1 methylation levels from BeadChip data, we first identified all the BeadChip’s CpGs lying in LINE-1 sequences according to the UCSC Genome Browser database. The cumulative DNA methylation level of LINE-1 sequences was computed, for each subject, as the average methylation level across the 12,762 CpGs, out of the >450K assayed on the BeadChip, that were annotated in LINE-1 sequences. Case-control differences were assayed by logistic regression, with methylation levels as a continuous variable and the same adjustment used for the case-control DMR discovery and replication analyses. For replication purposes, the same analysis was performed on the EPIC-NL samples using LINE-1 methylation data from MassARRAY analysis (Additional file 1). A LINE-1 methylation meta-analysis of the two studies was also done as described above.

### DNA methylation and MI risk

EPICOR and EPIC-NL subjects, stratified by sex and by study, were clustered with a RPMM algorithm [58] into four classes according to their *ZBTB12*-DMR methylation profile, irrespective of case-control status. Each subject was also allocated to a LINE-1 methylation class (above/below the median). The association between MI and DNA methylation (as RPMM class or LINE-1 methylation profile) was evaluated on the EPIC-NL panel by logistic regression analysis, stratifying by sex.

Moreover, to test the dependence/independence of the DNA methylation effects from the TRFs, we compared the ORs associated to each RPMM class and to LINE-1 methylation status under three logistic regression models, progressively including additional covariates at each step. To this purpose, the same analysis was done on the EPICOR discovery panel as well, under the caveat that the estimated ORs in this case should not be considered as a risk estimate, being assessed in the discovery panel and, as such, putatively inflated. Briefly, model 1 included the matching variables only, model 2 included the whole set of covariates used for the case-control DMR discovery and replication analyses, and model 3 was fully adjusted with the comprehensive set of variables as available in the two studies. Further methodological details are provided in Additional file 1.

### Discrimination, reclassification, and calibration

We tested for the improvement in the performance of MI risk prediction when including DMR and LINE-1 profiles identified in the EPICOR dataset (discovery phase) by running discrimination and reclassification analyses on the independent EPIC-NL dataset. Two models were compared: the first one included only TRFs that were significantly associated to MI in our study or reported in the literature to be associated to MI (Fig. 1, legend); the second one comprised TRFs as model 1 plus *ZBTB12*-RPMM classes and LINE-1 methylation class.

For discrimination, we compared the AUC of the two models by the DeLong test [59]. For reclassification, we computed the NRI and IDI indices [60]. The goodness of fit was evaluated by the Hosmer-Lemeshow (HL) test [61] in order to assess the proper calibration of the model.

### DNA methylation and TTD

Being EPICOR and EPIC-NL prospective cohorts with incident MI cases identified during cohort follow-up, we investigated the relationship between methylation and TTD, i.e., the time lapse between blood collection and the MI event. EPICOR and EPIC-NL cases, stratified by study and by sex, were divided in tertiles according to TTD. Control groups were used as reference. The occurrence of a linear trend between DNA methylation levels and TTD, as the ordinal categorical variable, was tested by GEE (details in Additional file 1).

## Additional files

Additional file 1: **Supplementary methods.** A document with supplementary materials, including the following: (1) subjects: cohort details; lifestyle, anthropometrics, and biochemical measurements; and outcome definition; (2) laboratory methods: EPICOR sample preparation; discovery phase: Illumina Human450K Methylation Assay; replication phase on EPIC-NL sample: Sequenom MassARRAY; and (3) supplementary statistical methods: case-control differential methylation; removal of technical biases; DNA methylation and MI risk; DNA methylation and time to disease (TTD); supplementary references. (DOCX 73 kb)

Additional file 2: **Supplemental Tables S1, S2, S3, and S4.** **Table S1.** top 6 genic DMRs in EPICOR MI overall cases vs controls. **Table S2A.** details of the ZBTB12-DMR CpGs in EPICOR subjects. **Table S2B.** details of the *ZBTB12*-DMR CpGs in EPIC-NL subjects. **Table S3A.** EPIC-NL MI risk, adjusted models. **Table S3B.** EPICOR, adjusted models. **Table S4A.** EPICOR case-control differential methylation analysis: comparison of models with and without lipids adjustment. **Table S4B.** EPIC-NL case-control differential methylation analysis: comparison of models with and without lipids, batch, and WBCs adjustments. (DOCX 55 kb)

Additional file 3: **Supplemental Figures S1, S2, S3, and S4.** **Figure S1.** quantile-quantile plot, EPICOR overall subjects. **Figure S2.** quantile-quantile plot, EPICOR men. **Figure S3.** quantile-quantile plot, EPICOR women. **Figure S4.** locations of *ZBTB12* and LINE-1 CpG sites investigated by Sequenom MassARRAY. CpGs (in red) investigated within ZBTB12-DMR, LINE-1, and flanking primers (upper case: complementary to DNA; lower case: T7-promoter sequence and 10mer tag). CpG sites that could not be tested individually due to MassARRAY technology constrains, but had to be tested jointly with neighboring CpGs as a single unit, are underlined: the methylation level is the cumulative value of all the sites within the CpG unit. (ZIP 91 kb)

## Abbreviations

AUC: area under the receiver operating curve
Beta-value: estimate of methylation level at each CpG
BMI: body mass index
CVD: cardiovascular disease
DMR: differentially methylated region
EPIC: European Prospective Investigation into Cancer and Nutrition
EWAS: epigenome-wide association study
FDR: false discovery rate
GEE: generalized estimating equations
IDI index: integrated discrimination improvement index
LINE-1: long interspersed nuclear element-1
MBPs: methyl-CpG binding proteins
MI: myocardial infarction
NRI index: net reclassification improvement index
QC: quality control
RPMM: recursively partitioned mixture model
TRFs: traditional risk factors
TTD: time to disease
WBCs: white blood cells
WHR: waist-to-hip ratio
