# Supplementary material for: Gene-specific DNA methylation profiles and LINE-1 hypomethylation are associated with myocardial infarction risk
Source: Clin Epigenetics. 2015 Dec 24;7:133. doi: 10.1186/s13148-015-0164-3 (PMC4690365; doi:10.1186/s13148-015-0164-3)
Supplement: Supplementary file 1 — Supplementary methods. A document with supplementary materials, including the following: (1) subjects: cohort details; lifestyle, anthropometrics, and biochemical measurements; and outcome definition; (2) laboratory methods: EPICOR sample preparation; discovery phase: Illumina Human450K Methylation Assay; replication phase on EPIC-NL sample: Sequenom MassARRAY; and (3) supplementary statistical methods: case-control differential methylation; removal of technical biases; DNA methylation and MI risk; DNA methylation and time to disease (TTD); supplementary references. (DOCX 73 kb) [file 13148_2015_164_MOESM1_ESM.docx]

**ADDITIONAL FILE 1: Supplementary Methods**

**Guarrera S., *et al.* Gene-specific DNA-methylation profiles and LINE-1 hypomethylation are associated with myocardial infarction risk**

**Subjects: Cohort details**

All the study subjects were recruited within the EPIC cohort [[1](#_ENREF_1)], that comprises ≈500,000 healthy volunteers recruited between 1993-1998 in 10 European countries, who provided at enrollment a detailed dietary and lifestyle questionnaire, as well as a blood sample that was stored in liquid nitrogen until use. The EPIC cohort is regularly followed up for events, such as occurrence of cancers and other non-communicable diseases of adulthood. The EPIC-Italy cohort (≈50,000 participants) [[2](#_ENREF_2)] was recruited between 1992 and 1998 in 4 Italian centers: Turin and Varese (Northern Italy), Florence (Central Italy) and Ragusa (Southern Italy), with the later inclusion of Naples (Central-Southern Italy), who provided additional samples, all females, belonging to the ATENA Study [[3](#_ENREF_3)] on cardiovascular disease and cancer. Follow-up of the cohort is regularly ongoing, with the last cardiovascular follow-up updated to 2010.

The **EPICOR study** [[4](#_ENREF_4)] is a case-cohort study nested within the EPIC-Italy cohort: 4 EPIC-Italy centers (Turin, Varese, Naples, and Ragusa) provided samples to EPICOR.

EPICOR comprises more than 1,500 subjects with cardiovascular outcomes, including myocardial infarction (MI), acute coronary syndrome, ischemic cardiomyopathy, coronary or carotid revascularization, ischemic- or hemorrhagic stroke, and aims to investigate dietary- and lifestyle-related cardiovascular risk factors.

The **EPIC-Netherlands cohort** [[5](#_ENREF_5)] (EPIC-NL) comprises the Prospect-EPIC and the Monitoring Project on Risk Factors for Chronic diseases (MORGEN) cohorts, that were set up simultaneously in 1993-1997 and recruited a total of 40,011 participants. The Prospect-EPIC study included 17,357 women aged 49-70 years, living in Utrecht and vicinity, who participated in a nationwide breast cancer screening program. The MORGEN-EPIC cohort consists of 22,654 men and women aged 20-65 years selected from random samples of the Dutch population in three Dutch towns (Doetinchem, Amsterdam and Maastricht).

In the EPIC cohort the prevalence of diabetes at enrolment was recorded through the administered questionnaires, and incident diabetes cases were identified at cohort regular follow-up. Subjects with prevalent diabetes were excluded from our study, as the purpose of our study was to identify epigenetic risk factors potentially independent from the already known strong CVD risk factors, such as diabetes.

*Lifestyle, anthropometrics, and biochemical measurements*

For the EPICOR subjects, information on reproductive history, physical activity, smoking history, alcohol consumption, medical history, occupation, educational level and other socio-economic variables was collected through self-administered questionnaires (specifically printed in two versions for men and women) compiled by each subject at enrollment into EPIC-Italy [[2](#_ENREF_2)], together with a detailed food-frequency questionnaire [[6](#_ENREF_6)]. Anthropometric parameters (weight, height, sitting height, waist and hip circumferences) and blood pressure were measured at enrollment according to standardized protocols [[2](#_ENREF_2)]. Body mass index (BMI) was calculated as weight divided by height squared (kg/m2); waist-to-hip ratio (WHR) was calculated as the ratio of the circumference of the waist to that of the hips.

For the EPIC-NL subjects, information on demographic characteristics, presence of chronic diseases and related potential risk factors was obtained with the general questionnaire at baseline. During the physical examination at baseline body weight, height and waist circumference were measured. BMI was calculated as weight divided by height squared (kg/m2). Mean systolic and diastolic blood pressure were obtained by calculating the mean of two subsequent measurements which were performed in supine position with a cuff on the left arm using either a Boso Oscillomat (Bosch & Son, Jungingen, Germany) (Prospect) or a random zero sphygmomanometer (MORGEN). Hypertension was considered present when at least one of the following criteria were met: systolic blood pressure >140 mmHg, diastolic blood pressure >90 mmHg, self-reported use of antihypertensive medication use, or self-report of physician-diagnosed hypertension. Total cholesterol levels were measured using enzymatic methods and high-density lipoprotein (HDL)-cholesterol and low-density lipoprotein (LDL)-cholesterol were measured using a homogeneous assay with enzymatic endpoint. These assays were all performed on an autoanalyzer (LX20, Beckman Coulter, Mijdrecht, the Netherlands).

*Outcome definition*

For the EPICOR sample, the ascertainment of vital status was carried out through linkage with demographic rosters. EPICOR MI cases were identified from hospital discharge databases when the clinical record reported the International Classification of Diseases (ICD), Ninth Revision, Clinical Modification code 410, or ICD 410 plus the procedure codes for coronary revascularization (e.g. percutaneous trans-luminal coronary angioplasty and coronary artery bypass surgery).

Suspect CHD events were confirmed when acute myocardial infarction, acute coronary syndrome, or coronary revascularization were reported in the records, supported by information on onset symptoms, levels of cardiac enzymes and troponins, and electrocardiographic data coded according to the Minnesota Code. Cases were cross-checked with mortality files to identify fatal and nonfatal cases (the latter defined as alive 28 days after diagnosis). Study participants with CHD at cohort entry were identified from the baseline questionnaire, from linkage with hospital discharge records, or by direct examination of clinical records, and were excluded from this study.

For EPIC-NL, morbidity data were obtained from the Dutch Center for Health Care Information, which holds a standardized computerized register of hospital diagnoses. Admission files from general and university hospitals in the Netherlands have been stored continuously since 1990. The records contain data on sex, date of birth, dates of admission and discharge, at least one principal diagnosis and up to nine optional additional diagnoses. All events were coded by qualified medical administrative personnel in the hospitals, according to the International Classification of Diseases, Ninth Revision, Clinical Modification (ICD-9). The National Medical Registry checked the data and collected them in the Hospital discharge Diagnosis Database, which is linked to the cohort based on information of birth date, sex, postal code and general practitioner with a validated probabilistic method [[7](#_ENREF_7)]. Information on vital status was obtained through digital linkage with municipal registries and causes of death were obtained through linkage with Statistics Netherlands. We identified all first ever MI (ICD-9; 410) events. Follow up was complete until January 1st 2006.

***Laboratory methods***

*EPICOR Sample preparation*

Genomic DNA was extracted from 400ul buffy coat from whole blood (stored in liquid nitrogen at sample recruitment) by an automated on-column DNA purification method using a QIAsymphony instrument with dedicated QIAsymphony DNA Kits (QIAGEN GmbH, Germany), according to manufacturer’s standard protocols. DNA integrity was checked by an electrophoretic run in standard TBE 0.5X buffer on a 1% low melting agarose gel (Sigma-Aldrich GmbH, Germany); DNA purity and concentration were assessed by a NanoDrop 8000 Spectrophotometer (Thermo Fisher Scientific Inc.).

*Discovery phase on the EPICOR sample: Illumina Human450K Methylation Assay*

The EZ-96 DNA Methylation-Gold Kit (Zymo Research Corporation) was used for the bisulfite conversion of 500ng of genomic DNA for each sample, according to manufacturer’s standard protocol. The methylation status can be assessed by distinguishing between a C or T residue at the same position in the bisulfite treated DNA [[8](#_ENREF_8)].

The Infinium HumanMethylation450 BeadChip (Illumina Inc., San Diego, CA, USA) was used to assess the methylation status of more than 485,000 individual CpG loci at a genome-wide resolution, including CpG islands, CpG sites outside of CpG islands (island shores and shelves, gene body), tumor differentially methylated regions and stem cell reprogramming differentially methylated regions [[9](#_ENREF_9)]. The assay covers 21,231 genes (98.9% of UCSC genes) and 29,249 transcripts not included in UCSC database, providing semi-quantitative methylation data with an average of 17.2 probes per gene region. The Infinium HumanMethylation450 BeadChip uses both Infinium I and II technology, relying on hybridization of enzymatically fragmented bisulfite converted DNA to locus specific DNA oligomers, followed by a single base extension reaction with Biotin- or DNP-labeled ddNTPs, that allows the discrimination between methylated (unconverted) or unmethylated (converted) cytosines at each CpG site. After fluorescent staining, the BeadChips were imaged with a dedicated scanner and fluorescent signals recoded. The average methylation value at each locus, or average “Beta-value”, was computed with the dedicated GenomeStudio Methylation software (v2011.1, llumina Inc., San Diego, CA) as the ratio of the intensity of the methylated signal over the total signal (unmethylated + methylated) [[10](#_ENREF_10), [11](#_ENREF_11)]. Thus, Beta-values represent the percentage of methylation at each individual CpG locus, ranging from 0 to 1.

All the samples were analyzed in 8 consecutive bisulfite conversion batches. Cases and controls were randomly and blindly distributed across conversion plates and methylation BeadChips. Twelve samples were analyzed on each BeadChip. As a “position effect” was reported for Illumina Methylation BeadChips, each sample position on the BeadChip was completely random as well. We further verified the randomization of the position on each BeadChip was effective by checking for a position effect, and we found no occurrence of position effect (data not shown).

The raw data were quality checked according to the built in quality controls, i.e. more than 200 control probes specifically designed to assess technical aspects of the bisulfite conversion process and of the BeadChip assay itself, including Bisulfite Conversion, Normalization (system background), Staining efficiency, Extension efficiency, Hybridization efficiency, Target Removal (stripping after extension reaction), Specificity.

Whole-genome methylation data quality control (QC) and normalization procedures are detailed in the main paper.

*Replication phase on EPIC-NL sample: Sequenom MassARRAY*

One aliquot of EPIC-NL subjects’ DNA was provided by the Utrecht centre.

MALDI-TOF mass spectrometry (MassARRAY EpiTYPER technology, Sequenom) [[12](#_ENREF_12)] was used for the quantitative assessment of the DNA methylation levels of the genomic region significantly differentially methylated in cases vs. controls (DMR) from the EPICOR dataset, as well as for LINE-1 methylation analysis.

DNA bisulfite conversion was performed by the EZ-96 DNA Methylation Gold Kit (Zymo Research) according to the manufacturer protocol.

Bisulfite-specific primers for the PCR reactions were designed with the EpiDesigner Tool. PCR products were SAP-digested, cleaved, and purified according to the MassARRAY EpiTyper Assay standard protocol. Samples were analyzed on 384-well plates in 4 consecutive batches. MassARRAY data were visualized with MassARRAY EpiTyper v1.0 software.

Bisulfite conversion efficiency for each sample was evaluated by the specific R based tool MassArray, and all samples scored the highest (100% conversion efficiency).

Sixteen EPICOR samples previously analyzed with the HumanMethylation450 BeadChip were also blindly analyzed for comparison and validation purposes.

*ZBTB12 MassARRAY Methylation Analysis*: due to assay design constrains, only a subset of the CpGs assayed by the Illumina BeadChip could be tested with the MassARRAY assay. This is due to the fact that the technique itself uses a multiplex PCR followed by mass spectrometry analysis of generated fragments. The assay should be thus designed so not to have cross reactions of PCR probes and overlapping fragment for mass spectrometry analysis: depending on the context sequence, this makes several CpGs not analyzable by MassARRAY. For the same reason, the MassARRAY assay is unable to discriminate between CpGs located at close vicinity to each other in the sequence: in this case, the close neighboring CpGs are analyzed as “Units”, i.e. the measured methylation level is the average of the methylation levels of the CpGs cumulatively analyzed within the Unit.

On the other side, in the replica panel we could analyze by MassARRAY several CpGs in the *ZBTB12*-DMR that were not included in the Illumina BeadChip probe-set. Thirty two CpG sites within the *ZBTB12*-DMR that complied with Sequenom MassARRAY design requirements were selected and analyzed as described above. Locations of CpGs within *ZBTB12* region and flanking primers are detailed in Supplementary Figure S4.

A total of 30 CpGs, corresponding to 23 methylation signals, were included in the analysis, except for CpG_12 and CpG_32 that showed no signal. The A-clustering [[13](#_ENREF_13)] analysis of correlated methylation levels (see Supplementary statistical methods, below) identified CpG_31 as outsider from the cluster of CpGs with correlated methylation, and thus CpG_31 was excluded from further analyses.

To test for the reproducibility of the methylation levels measured with the two methods, we challenged with the MassARRAY 16 samples already analyzed with the Illumina BeadChip. Nine of the CpGs analyzed with the BeadChip (i.e. cg16463880, cg00459243, cg14734916, cg06636203, cg09788778, cg25470384, cg25861453, cg17243044, cg00058449) could be analyzed as single CpG also with the MassARRAY, and their methylation values were used to check the reproducibility between the 2 techniques. The mean BeadChip methylation value of all the 9 CpGs, and the corresponding mean MassARRAY methylation value of the same CpGs were computed for each sample, and tested for differences. Correlation was found between the 2 series of measures (Pearson R = 0.56, p = 0.02). Moreover, the average intra-individual coefficient of variation (0.07) is smaller than the inter-individual coefficient of variation (0.09).

*LINE-1 MassARRAY methylation analysis*: we investigated LINE-1 methylation profiles through the targeted analysis of 11 CpGs, according to Wang *et al*. [[14](#_ENREF_14)].

Locations of CpGs within LINE-1 region and flanking primers are detailed in Supplementary Figure S4.

A total of 11 CpGs, corresponding to 8 methylation signals (5 single CpGs and 3 CpG-Units), were included in the analysis, except for CpG_10 that returned no signal.

The 11 CpGs, being part of a LINE-1 sequence, are represented thousands of times in the genome, as LINE-1 sequences are very abundant and ubiquitous across the genome. The methylation levels of each of the 8 methylation signals (single CpG or CpG-unit for closely neighboring CpGs) is thus the average value of all the corresponding CpGs (with the same sequence context) scattered across the genome, and the methylation level of each of the 8 methylation signals likely reflects the mean methylation level of that position in all the thousands of LINE-1 sequences within the genome. The 8 signals were then averaged to obtain a mean value that we used as cumulative mean methylation value of LINE-1 sequences across the genome.

***Supplementary statistical methods***

Several analytical approaches were used in this study to assess and verify under different aspects the DNA methylation effects on MI risk.

*Case-control differential methylation*

The Adjacent Sites Algorithm (A-clustering, or shortly Aclust) by Sofer *et al* [[13](#_ENREF_13)] was used on EPICOR epigenome-wide DNA-methylation data to delimit genetic regions by identifying clusters of CpGs with correlated methylation levels. In brief, Aclust investigates the correlation structure of adjacent CpG sites to identify regions with neighboring CpG sites exhibiting common behavior. Unlike other methods, that detect regions of differential methylation right at the initial step (e.g. sliding-window and/or bump-hunting based methods), with Aclust stretches of consecutive CpGs are scanned for coordinated methylation independently from the occurrence of differential methylation. Genetic regions are delimited and tagged as “clusters of coordinated methylation” every time a stretch of continuous CpGs with coordinated methylation is seen, by clustering together neighboring CpG sites according to their correlation.

The regions of correlated methylation identified in the EPICOR discovery panel were then tested by Generalized Estimating Equations (GEE) [[15](#_ENREF_15)] to check the occurrence of differential methylation between MI cases and controls. GEE method works well with correlated data, such as it is the case with the clusters of correlated CpGs we analyzed in our study. Aclust CpGs clustering and GEE methods were used for the analyses of both the EPICOR discovery panel and the EPIC-NL replica panel.

For the DMR analysis, GEE were used with a minimal set of covariates (see main paper) in order to reduce the “statistical noise” in the model that may derive from an excess of degrees of freedom.

Lipid profile (HDL cholesterol, LDL cholesterol, triglycerides) was not included in our case-control DMR analysis as 48 EPICOR subjects and 25 EPIC-NL subjects had missing information. We nevertheless verified in the EPICOR and EPIC-NL panels that the exclusion of this variable did not affect the analysis itself. To do so, we compared the effect-sizes of *ZBTB12*-DMR and LINE-1 methylation in the models with and without lipid profile as covariate. Differences between effect-sizes with and without lipids in the model were tested by computing the standard error (se) of the effect-size difference as $se=\sqrt{\mathrm{se}_{1}^{2}+\mathrm{se}_{2}^{2}}$where se_1_ is the standard error of effect-size_1_ and se_2_ is the standard error of effect-size _2_, and then comparing the statistic $Z=\frac{effect.size1- effect.size 2}{\mathrm{se}}$ with a standard Gaussian distribution to compute *P*-values. *P*-values>0.05 indicate no difference in the effect-size estimate. As no statistically significant difference was found between the lipids corrected and the lipids non-corrected estimated effect-sizes (see Additional File 1, Table S4A and B), we omitted the lipid profile correction in the DMR analyses of both the EPICOR and EPIC-NL panels.

The same comparison was made for the inclusion/exclusion of White Blood Cells (WBCs) count as covariate in the EPIC-NL analyses. For the EPICOR sample the percentage of WBCs subtypes was estimated from genome-wide methylation data [[16](#_ENREF_16)] for each subject and used as covariate in the differential methylation analyses; for the EPIC-NL sample, where targeted DNA-methylation analysis was performed, no such estimate was feasible, due to the unavailability of genome-wide data. Since WBCs count was available for a subset of subjects (N=204, 35% of the NL sample), we compared the models including or excluding WBC in the *ZBTB12*-DMR and LINE-1 analyses of EPIC-NL sample. We found no statistically significant difference between the WBCs corrected and the non-WBCs corrected estimated effect-sizes (see Additional File 1, Table S4B), and thus WBCs count was not used as covariate in the EPIC-NL analyses.

To account for methylation assays variability and batch effects, we corrected GEE analyses of EPICOR sample for control probes Principal Components (PCs), while for EPIC-NL no batch correction was needed (see the next paragraph).

*Removal of technical biases*

For the EPICOR sample, the methylation levels were assessed by the Illumina 450K methylation array, which includes built-in control probes (see *Discovery phase: Illumina Human450K Methylation Assay* paragraph above). Lehne *et al*. showed that the PCs assessed by principal a component analysis (PCA) of control probes intensities correlated closely with multiple technical parameters, including bisulfite conversion batch and plates [[17](#_ENREF_17)]. We thus included control probes PCs in the EPICOR GEE analysis, progressively incrementing the number of PCs until no significant residual statistical inflation was found. Adjustment for the first 20 PCs almost entirely removed statistic inflation, consistent with effective correction for batch and technical effects (inflation lambda <1.05).

EPIC-NL subjects were analyzed in 11 consecutive bisulfite conversion batches, and 4 consecutive Sequenom runs. The same “control” sample was included in each conversion batch, and each of the so converted controls were run on the Sequenom along with the other samples of the same conversion batch. We measured the average variation coefficient of the methylation measurements across all the tested CpG sites for the control sample (Mean CV= 0.047) and for all the target samples (Mean CV= 0.233). As the mean variation coefficient within repeated measurements of the same control sample is 5X less than the inter-individual variation coefficient, we could assume that batch effect did not significantly change the estimated differences between cases and controls. To further verify this assumption, we reanalyzed the EPIC-NL data by including into the DMR analyses also the conversion batch (11 classes) and the Sequenom run (4 classes), and found comparable effect-sizes (see Additional File 1, Table S4B). As the effect-size due to methylation did not change, we can assume no effect of “batch” correction on the analysis results. Batch correction was thus omitted in the analysis of EPIC-NL methylation data.

*DNA methylation and MI risk*

To evaluate the risk associated to differential methylation of *ZBTB12*-DMR and LINE-1, each subject was allocated to classes according to her/his *ZBTB12*-DMR and LINE-1 methylation profile, respectively.

The RPMM algorithm, that proved to be suitable and robust to analyze data produced with the Illumina HumanMethylation450 BeadChip [[18](#_ENREF_18), [19](#_ENREF_19)], was used to cluster subjects according to their *ZBTB12*-DMR profile, irrespective of case-control status, allowing the estimation of the MI risk associated to the overall region profile.

Subjects were also categorized according to their LINE-1 methylation levels as being above or below the median value of the group. This categorization was done to account for the different methods used to measure LINE-1 methylation levels: for the EPICOR sample, more than 1000 single CpGs values across the genome were averaged to achieve an estimate of the overall LINE-1 methylation, whilst for the EPIC-NL panel the measurements were done by MassArray by measuring 8 methylation signals (5 single CpGs and 3 CpG units) which are nevertheless present as multiple copies scattered across the genome, being LINE-1 sequence specific CpGs. Under these conditions, the RPMM clustering was not properly suitable to classify subjects, while the above/below the median cutoff provided a good option to compare measurements achieved with such different methods.

After allocating each subject to a RPMM class and to a LINE-1 class, we estimated, in the EPIC-NL panel, the MI risk associated to the different DNA-methylation profiles. Three different logistic regression models were used for the EPIC-NL sample: Model 1, adjusting for age and center of recruitment; Model 2, as Model 1 plus smoke, BMI, blood pressure; Model 3, as Model 2 plus WHR, alcohol, LDL, HDL, triglycerides, menopausal status in women. As we found lower methylation levels in MI cases, the class with the highest methylation (RPMM-0 and LINE-1 methylation above the median) was taken as the reference (results in Additional file 1, Table S3A).

The progressive inclusion of additional covariates (i.e. additional CAD risk factors) was done with the purpose to verify that: 1) the inclusion of further covariates did not affect the estimated association between DMRs’ DNA methylation levels and MI risk, and 2) that the risk associated to *ZBTB12*-DMR and LINE-1 methylation levels was indeed independent form that conferred by other risk factors.

To verify the independency of DNA-methylation profiles from the traditional risk factors (TRFs) in the discovery panel too, the same analysis was done on the EPICOR panel. It is due to underline that, as stated in the main paper, in this case the ORs cannot be considered as an estimate of risk.

Three logistic regression models were used for the analysis of the EPICOR sample: Model 1, adjusting for matching variables; Model 2, comprised the whole set of variables included in the DMR discovery, i.e. matching variables, smoking status, BMI, physical activity, blood pressure, estimated WBC count, control probes PCs; Model 3, as Model 2 plus WHR, alcohol, LDL, HDL, triglycerides, economical index, glucose lowering medical treatments, menopausal status in women (results in Additional file 1, Table S3B).

Differences between ORs from the nested models (reported in Additional file 1, Tables S3A and B) were tested by computing the standard error (se) of the log(OR) difference as $se=\sqrt{\mathrm{se}_{1}^{2}+\mathrm{se}_{2}^{2}}$where se_1_ is the standard error of log(OR_1_) and se_2_ is the standard error of log(OR_2_), and then comparing the statistic $Z=\frac{log(\mathrm{OR}_{1} )- log(\mathrm{OR}_{2} )}{\mathrm{se}}$ with a standard Gaussian distribution to compute *P*-values.

*DNA-methylation and time to disease (TTD)*

Since our subjects panels are case-control studies (EPICOR samples are even individually matched), the investigated sample is enriched in cases (events) with respect to general population, making Poisson regression and Cox regression not appropriate. Under these conditions, GEE method was a good method of choice to test the occurrence of a linear trend between DNA-methylation levels and TTD as ordinal categorical variable. Subjects, stratified by study and by sex, were divided in tertiles (i.e. three groups of equal numerosity), and control groups were used as reference.

**Supplementary References**

1. Riboli E, Kaaks R. The EPIC Project: rationale and study design. European Prospective Investigation into Cancer and Nutrition. Int J Epidemiol. 1997;26 Suppl 1:S6-14.

2. Palli D, Berrino F, Vineis P, Tumino R, Panico S, Masala G et al. A molecular epidemiology project on diet and cancer: the EPIC-Italy Prospective Study. Design and baseline characteristics of participants. Tumori. 2003;89(6):586-93.

3. Panico S, Dello Iacovo R, Celentano E, Galasso R, Muti P, Salvatore M et al. Progetto ATENA, a study on the etiology of major chronic diseases in women: design, rationale and objectives. Eur J Epidemiol. 1992;8(4):601-8.

4. Bendinelli B, Masala G, Saieva C, Salvini S, Calonico C, Sacerdote C et al. Fruit, vegetables, and olive oil and risk of coronary heart disease in Italian women: the EPICOR Study. Am J Clin Nutr. 2011;93(2):275-83. doi:10.3945/ajcn.110.000521.

5. Beulens JW, Monninkhof EM, Verschuren WM, van der Schouw YT, Smit J, Ocke MC et al. Cohort profile: the EPIC-NL study. Int J Epidemiol. 2010;39(5):1170-8. doi:10.1093/ije/dyp217.

6. Pisani P, Faggiano F, Krogh V, Palli D, Vineis P, Berrino F. Relative validity and reproducibility of a food frequency dietary questionnaire for use in the Italian EPIC centres. Int J Epidemiol. 1997;26 Suppl 1:S152-60.

7. Herings RM, Bakker A, Stricker BH, Nap G. Pharmaco-morbidity linkage: a feasibility study comparing morbidity in two pharmacy based exposure cohorts. J Epidemiol Community Health. 1992;46(2):136-40.

8. Herman JG, Graff JR, Myohanen S, Nelkin BD, Baylin SB. Methylation-specific PCR: a novel PCR assay for methylation status of CpG islands. Proc Natl Acad Sci U S A. 1996;93(18):9821-6.

9. Bibikova M, Barnes B, Tsan C, Ho V, Klotzle B, Le JM et al. High density DNA methylation array with single CpG site resolution. Genomics. 2011;98(4):288-95. doi:10.1016/j.ygeno.2011.07.007.

10. Bibikova M, Lin Z, Zhou L, Chudin E, Garcia EW, Wu B et al. High-throughput DNA methylation profiling using universal bead arrays. Genome Res. 2006;16(3):383-93. doi:10.1101/gr.4410706.

11. Du P, Zhang X, Huang CC, Jafari N, Kibbe WA, Hou L et al. Comparison of Beta-value and M-value methods for quantifying methylation levels by microarray analysis. BMC Bioinformatics. 2010;11:587. doi:10.1186/1471-2105-11-587.

12. Ehrich M, Nelson MR, Stanssens P, Zabeau M, Liloglou T, Xinarianos G et al. Quantitative high-throughput analysis of DNA methylation patterns by base-specific cleavage and mass spectrometry. Proc Natl Acad Sci U S A. 2005;102(44):15785-90. doi:10.1073/pnas.0507816102.

13. Sofer T, Schifano ED, Hoppin JA, Hou L, Baccarelli AA. A-clustering: a novel method for the detection of co-regulated methylation regions, and regions associated with exposure. Bioinformatics. 2013;29(22):2884-91. doi:10.1093/bioinformatics/btt498.

14. Wang L, Wang F, Guan J, Le J, Wu L, Zou J et al. Relation between hypomethylation of long interspersed nucleotide elements and risk of neural tube defects. Am J Clin Nutr. 2010;91(5):1359-67. doi:10.3945/ajcn.2009.28858.

15. Liang KY, Zeger SL. Longitudinal Data-Analysis Using Generalized Linear-Models. Biometrika. 1986;73(1):13-22. doi:DOI 10.1093/biomet/73.1.13.

16. Houseman EA, Accomando WP, Koestler DC, Christensen BC, Marsit CJ, Nelson HH et al. DNA methylation arrays as surrogate measures of cell mixture distribution. BMC Bioinformatics. 2012;13:86. doi:10.1186/1471-2105-13-86.

17. Lehne B, Drong AW, Loh M, Zhang W, Scott WR, Tan ST et al. A coherent approach for analysis of the Illumina HumanMethylation450 BeadChip improves data quality and performance in epigenome-wide association studies. Genome Biol. 2015;16:37. doi:10.1186/s13059-015-0600-x; s13059-015-0600-x [pii].

18. Koestler DC, Marsit CJ, Christensen BC, Karagas MR, Bueno R, Sugarbaker DJ et al. Semi-supervised recursively partitioned mixture models for identifying cancer subtypes. Bioinformatics. 2010;26(20):2578-85. doi:10.1093/bioinformatics/btq470; btq470 [pii].

19. Houseman EA, Christensen BC, Yeh RF, Marsit CJ, Karagas MR, Wrensch M et al. Model-based clustering of DNA methylation array data: a recursive-partitioning algorithm for high-dimensional data arising as a mixture of beta distributions. BMC Bioinformatics. 2008;9:365. doi:10.1186/1471-2105-9-365.
