# Supplementary material for: Gene-specific DNA methylation profiles and LINE-1 hypomethylation are associated with myocardial infarction risk
Source: Clin Epigenetics. 2015 Dec 24;7:133. doi: 10.1186/s13148-015-0164-3 (PMC4690365; doi:10.1186/s13148-015-0164-3)
Supplement: Supplementary file 2 — Supplemental Tables S1, S2, S3, and S4. Table S1. top 6 genic DMRs in EPICOR MI overall cases vs controls. Table S2A. details of the ZBTB12-DMR CpGs in EPICOR subjects. Table S2B. details of the ZBTB12-DMR CpGs in EPIC-NL subjects. Table S3A. EPIC-NL MI risk, adjusted models. Table S3B. EPICOR, adjusted models. Table S4A. EPICOR case-control differential methylation analysis: comparison of models with and without lipids adjustment. Table S4B. EPIC-NL case-control differential methylation analysis: comparison of models with and without lipids, batch, and WBCs adjustments. (DOCX 55 kb) [file 13148_2015_164_MOESM2_ESM.docx]

**ADDITIONAL FILE 2**

**Guarrera S, *et al.*: Gene-specific DNA-methylation profiles and LINE-1 hypomethylation are associated with myocardial infarction risk**

**Table S1.** Top 6 genic DMRs in EPICOR overall MI cases *vs* controls (A-clustering method)

| **Gene name** | **CHR** | **CpGs in cluster** | ***P-value*** | ***Q-value**** | **Effect-size** | **se** |
| --- | --- | --- | --- | --- | --- | --- |
| ***ZBTB12*** | **6** | **cg10917426, cg08975528, cg16463880, cg00459243, cg14734916, cg14562426, cg06636203, cg11645762, cg09788778, cg25470384, cg00805874, cg04603811, cg25861453, cg25013586, cg13127825** | **1.94x10^-7^** | **0.005** | **-0.019** | **0.004** |
| *C22orf25;MIR185* | 22 | cg23095729, cg00390484, cg00504285 | 4.16x10^-6^ | 0.053 | -0.017 | 0.004 |
| *FARP1* | 13 | cg22022001, cg11733266 | 1.75x10^-5^ | 0.148 | -0.006 | 0.001 |
| *PLEKHA1* | 10 | cg03142365, cg11287521, cg10717722, cg01246254, cg24266261 | 2.53x10^-5^ | 0.161 | 0.005 | 0.001 |
| *CBFA2T3* | 16 | cg08732950, cg02431972, cg05953373 | 3.31x10^-5^ | 0.174 | 0.022 | 0.005 |
| *TMEM39A* | 3 | cg12184221, cg07890553 | 4.47 x10^-5^ | 0.247 | 0.024 | 0.006 |

**FDR-adjusted P-value*

**Table S2 A.** Details of the *ZBTB12*-DMR CpGs in EPICOR subjects

|  |  |  |  |  | **EPICOR OVERALL** | | | **EPICOR MEN** | | | **EPICOR WOMEN** | | |
| --- | --- | --- | --- | --- | --- | --- | --- | --- | --- | --- | --- | --- | --- |
| **TargetID** | **Chr** | **GRCh37/hg19 position** | **Gene** | **UCSC RefGene Group** | ***P**** | **Effect-size** | **se** | ***P**** | **Effect-size** | **se** | ***P**** | **Effect-size** | **se** |
| cg10917426 | 6 | 31867698 | *ZBTB12* | 3'UTR | 0.0005 | -0.018 | 0.005 | 0.0027 | -0.020 | 0.007 | 0.5074 | -0.006 | 0.008 |
| cg08975528 | 6 | 31867700 | *ZBTB12* | 3'UTR | 0.0002 | -0.018 | 0.005 | 0.0005 | -0.022 | 0.006 | 0.6438 | -0.003 | 0.007 |
| cg16463880 | 6 | 31867707 | *ZBTB12* | Body | 0.0003 | -0.017 | 0.005 | 0.0005 | -0.021 | 0.006 | 0.6482 | -0.003 | 0.007 |
| cg00459243 | 6 | 31867726 | *ZBTB12* | Body | 2.70E-05 | -0.018 | 0.004 | 6.18E-05 | -0.022 | 0.006 | 0.8289 | -0.001 | 0.006 |
| cg14734916 | 6 | 31867747 | *ZBTB12* | Body | 3.51E-07 | -0.023 | 0.005 | 5.52E-06 | -0.028 | 0.006 | 0.2195 | -0.009 | 0.007 |
| cg14562426 | 6 | 31867757 | *ZBTB12* | Body | 3.31E-06 | -0.022 | 0.005 | 9.21E-06 | -0.028 | 0.006 | 0.6364 | -0.004 | 0.009 |
| cg06636203 | 6 | 31867788 | *ZBTB12* | Body | 9.92E-05 | -0.015 | 0.004 | 0.0004 | -0.018 | 0.005 | 0.3196 | -0.006 | 0.006 |
| cg11645762 | 6 | 31867819 | *ZBTB12* | Body | 2.68E-05 | -0.018 | 0.004 | 2.67E-05 | -0.024 | 0.006 | 0.4589 | -0.005 | 0.006 |
| cg09788778 | 6 | 31867822 | *ZBTB12* | Body | 0.0042 | -0.019 | 0.007 | 0.0074 | -0.022 | 0.008 | 0.7032 | -0.005 | 0.013 |
| cg25470384 | 6 | 31867836 | *ZBTB12* | Body | 3.30E-12 | -0.021 | 0.003 | 5.51E-13 | -0.027 | 0.004 | 0.0683 | -0.010 | 0.006 |
| cg00805874 | 6 | 31867847 | *ZBTB12* | Body | 0.0738 | -0.013 | 0.007 | 0.0437 | -0.018 | 0.009 | 0.8412 | -0.003 | 0.013 |
| cg04603811 | 6 | 31867873 | *ZBTB12* | Body | 0.0005 | -0.017 | 0.005 | 0.0003 | -0.023 | 0.006 | 0.6314 | -0.004 | 0.007 |
| cg25861453 | 6 | 31867906 | *ZBTB12* | Body | 3.04E-05 | -0.018 | 0.004 | 0.0006 | -0.021 | 0.006 | 0.0933 | -0.008 | 0.005 |
| cg25013586 | 6 | 31867915 | *ZBTB12* | Body | 1.67E-15 | -0.022 | 0.003 | 1.75E-14 | -0.027 | 0.004 | 0.0007 | -0.014 | 0.004 |
| cg13127825 | 6 | 31867919 | *ZBTB12* | Body | 5.55E-16 | -0.022 | 0.003 | 4.76E-12 | -0.024 | 0.003 | 0.0073 | -0.011 | 0.004 |

*Single CpG, MI cases *vs* controls

**Table S2 B.** Details of the *ZBTB12*-DMR CpGs in EPIC-NL subjects

|  |  |  |  |  | **EPIC-NL OVERALL** | | | **EPIC-NL MEN** | | | **EPIC-NL WOMEN** | | |
| --- | --- | --- | --- | --- | --- | --- | --- | --- | --- | --- | --- | --- | --- |
| **TargetID** | **Chr** | **GRCh37/hg19 position** | **Gene** | **Corresponding cg# on BeadChip** | ***P**** | **Effect-size** | **se** | ***P**** | **Effect-size** | **se** | ***P**** | **Effect-size** | **se** |
| CHR6_CpG_1 | 6 | 31868066 | *ZBTB12* | -- | 0.0001 | -0.014 | 0.004 | 0.2872 | -0.007 | 0.006 | 0.0025 | -0.018 | 0.006 |
| CHR6_CpG_2 | 6 | 31868050 | *ZBTB12* | -- | 7.85E-08 | -0.020 | 0.004 | 5.10E-05 | -0.026 | 0.007 | 0.0065 | -0.016 | 0.006 |
| CHR6_CpG_3.4 | 6 | 31868025/021 | *ZBTB12* | cg25110523  cg12484688 | 0.0039 | -0.011 | 0.004 | 0.3496 | -0.006 | 0.007 | 0.0160 | -0.014 | 0.006 |
| CHR6_CpG_5 | 6 | 31867995 | *ZBTB12* | cg07249939 | 0.2278 | -0.004 | 0.004 | 0.9029 | -0.001 | 0.007 | 0.2346 | -0.007 | 0.006 |
| CHR6_CpG_6 | 6 | 31867956 | *ZBTB12* | cg00058449 | 0.3253 | -0.004 | 0.004 | 0.2327 | -0.006 | 0.006 | 0.5077 | -0.004 | 0.006 |
| CHR6_CpG_7 | 6 | 31867945 | *ZBTB12* | cg17243044 | 7.04E-04 | -0.012 | 0.004 | 0.0004 | -0.024 | 0.007 | 0.2768 | -0.006 | 0.006 |
| CHR6_CpG_8 | 6 | 31867938 | *ZBTB12* | -- | 0.0390 | -0.008 | 0.004 | 0.3133 | -0.007 | 0.007 | 0.1301 | -0.009 | 0.006 |
| **CHR6_CpG_9.10** | **6** | **31867919/915** | ***ZBTB12*** | **cg13127825/cg25013586** | 0.0738 | -0.007 | 0.004 | 0.5180 | -0.004 | 0.006 | 0.1367 | -0.009 | 0.006 |
| **CHR6_CpG_11** | **6** | **31867906** | ***ZBTB12*** | **cg25861453** | 0.0003 | -0.014 | 0.004 | 0.5574 | -0.004 | 0.007 | 0.0006 | -0.020 | 0.006 |
| **CHR6_CpG_12** | **6** | **31867873** | ***ZBTB12*** | **cg04603811** | -- | -- | -- | -- | -- | -- | -- | -- | -- |
| **CHR6_CpG_13** | **6** | **31867853** | ***ZBTB12*** | **--** | 0.0952 | -0.006 | 0.004 | 0.2953 | -0.007 | 0.007 | 0.3371 | -0.005 | 0.006 |
| **CHR6_CpG_14.15** | **6** | **31867847/845** | ***ZBTB12*** | **cg00805874/--** | 0.0725 | -0.007 | 0.004 | 0.5264 | -0.004 | 0.007 | 0.1358 | -0.009 | 0.006 |
| **CHR6_CpG_16** | **6** | **31867836** | ***ZBTB12*** | **cg25470384** | 3.45E-08 | -0.021 | 0.004 | 9.10E-05 | -0.025 | 0.006 | 0.0035 | -0.017 | 0.006 |
| **CHR6_CpG_17** | **6** | **31867822** | ***ZBTB12*** | **cg09788778** | 0.0008 | -0.012 | 0.004 | 0.0004 | -0.024 | 0.007 | 0.2679 | -0.006 | 0.006 |
| **CHR6_CpG_18.19.20** | **6** | **31867819/815/810** | ***ZBTB12*** | **cg11645762/--/--** | 0.0003 | -0.014 | 0.004 | 0.0008 | -0.022 | 0.006 | 0.1202 | -0.009 | 0.006 |
| **CHR6_CpG_21** | **6** | **31867788** | ***ZBTB12*** | **cg06636203** | 8.39E-05 | -0.015 | 0.004 | 3.74E-09 | -0.039 | 0.007 | 0.9132 | 0.001 | 0.006 |
| **CHR6_CpG_22** | **6** | **31867768** | ***ZBTB12*** | **--** | 0.7438 | -0.001 | 0.004 | 0.0877 | 0.011 | 0.007 | 0.1429 | -0.009 | 0.006 |
| **CHR6_CpG_23.24** | **6** | **31867757/753** | ***ZBTB12*** | **cg14562426/--** | 0.0150 | -0.009 | 0.004 | 0.1297 | -0.010 | 0.007 | 0.1492 | -0.008 | 0.006 |
| **CHR6_CpG_25** | **6** | **31867747** | ***ZBTB12*** | **cg14734916** | 9.87E-05 | -0.014 | 0.004 | 0.2968 | -0.007 | 0.007 | 0.0026 | -0.018 | 0.006 |
| **CHR6_CpG_26** | **6** | **31867726** | ***ZBTB12*** | **cg00459243** | 6.91E-07 | -0.018 | 0.004 | 0.0161 | -0.016 | 0.007 | 0.0008 | -0.020 | 0.006 |
| **CHR6_CpG_27** | **6** | **31867717** | ***ZBTB12*** | **--** | 0.0601 | -0.007 | 0.004 | 0.9225 | 0.001 | 0.006 | 0.0534 | -0.011 | 0.006 |
| **CHR6_CpG_28** | **6** | **31867707** | ***ZBTB12*** | **cg16463880** | 1.26E-07 | -0.020 | 0.004 | 2.01E-05 | -0.028 | 0.007 | 0.0061 | -0.016 | 0.006 |
| **CHR6_CpG_29.30** | **6** | **31867700/698** | ***ZBTB12*** | **cg08975528/cg10917426** | 8.19E-05 | -0.015 | 0.004 | 2.09E-05 | -0.028 | 0.006 | 0.1864 | -0.008 | 0.006 |
| CHR6_CpG_31 | 6 | 31867692 | *ZBTB12* | -- | -- | -- | -- | -- | -- | -- | -- | -- | -- |
| CHR6_CpG_32 | 6 | 31867659 | *ZBTB12* | -- | -- | -- | -- | -- | -- | -- | -- | -- | -- |

*****Single CpG, MI cases *vs* controls

In bold the CpGs (11 single CpGs and 5 CpG-units) corresponding to the 15-CpGs cluster identified by A-clustering, which is differentially methylated between cases and controls in the EPICOR discovery panel. Corresponding cg# identifiers (i.e. Illumina BeadChip identifiers) are reported in the “Corresponding cg# on BeadChip” column.

**Table S3A.** EPIC-NL MI risk, Adjusted Models

|  |  | **MODEL 1** | | | **MODEL 2** | | | | | **MODEL 3** | | | | | |
| --- | --- | --- | --- | --- | --- | --- | --- | --- | --- | --- | --- | --- | --- | --- | --- |
| **EPIC-NL MEN** | ***P*_deltaOR_^*^** | **OR** | **95%CI** | ***P*** | **OR** | **95%CI** | | ***P*** | | | **OR** | **95%CI** | | ***P*** | |
| **RPMM-0[0.59] ^†^** |  | 1 | - | - | 1 | | - | | - | | 1 | | - | | - |
| **RPMM-1[0.55] ^†^** | 0.835 | 1.024 | 0.411-2.554 | 0.959 | 1.102 | | 0.424-2.866 | | 0.842 | | 1.185 | | 0.424-3.309 | | 0.746 |
| **RPMM-2[0.50] ^†^** | 0.602 | 1.115 | 0.423-2.940 | 0.826 | 1.263 | | 0.457-3.489 | | 0.653 | | 1.644 | | 0.552-4.899 | | 0.372 |
| **RPMM-3[0.49] ^†^** | 0.597 | 1.696 | 0.598-4.808 | 0.320 | 1.846 | | 0.619-5.505 | | 0.272 | | 2.599 | | 0.789-8.560 | | 0.116 |
| **LINE-1 methylation below the median** | 0.896 | 2.062 | 1.151-3.695 | 0.015 | 1.941 | | 1.069-3.523 | | 0.029 | | 1.946 | | 1.020-3.712 | | 0.043 |
|  |  |  |  |  |  | |  | |  | |  | |  | |  |
| **EPIC-NL WOMEN** | ***P*_deltaOR_^*^** | **OR** | **95%CI** | ***P*** | **OR** | | **95%CI** | | ***P*** | | **OR** | | **95%CI** | | ***P*** |
| **RPMM-0[0.60] ^†^** |  | 1 | - | - | 1 | | - | | - | | 1 | | - | | - |
| **RPMM-1[0.56] ^†^** | 0.820 | 1.528 | 0.811-2.878 | 0.189 | 1.579 | | 0.816-3.056 | | 0.175 | | 1.706 | | 0.844-3.448 | | 0.137 |
| **RPMM-2[0.52] ^†^** | 0.533 | 1.699 | 0.299-9.645 | 0.550 | 2.249 | | 0.385-13.125 | | 0.368 | | 3.829 | | 0.587-24.964 | | 0.160 |
| **RPMM-3[0.48] ^†^** | 0.681 | 2.272 | 1.238-4.171 | 0.008 | 2.350 | | 1.249-4.421 | | 0.008 | | 2.752 | | 1.389-5.450 | | 0.004 |
| **LINE-1 methylation below the median** | 0.778 | 0.956 | 0.627-1.456 | 0.833 | 1.037 | | 0.670-1.605 | | 0.872 | | 1.046 | | 0.655-1.670 | | 0.850 |
|  |  | Adj. by age and center of recruitment^‡^ | | | MODEL 1 + smoke, BMI, blood pressure | | | | | | MODEL 2 + WHR, alcohol, LDL, HDL, triglycerides, menopausal status in women | | | | |

^*^see Suppl. Methods, *DNA methylation and MI risk* paragraph

^†^Mean Methylation value in square brackets

^‡^Adjustment for center of recruitment was done only for EPIC-NL women, as men were all recruited in the same center.

**Table S3B.** EPICOR, Adjusted Models

|  |  | **MODEL 1** | | | **MODEL 2** | | | | | **MODEL 3** | | | | | |
| --- | --- | --- | --- | --- | --- | --- | --- | --- | --- | --- | --- | --- | --- | --- | --- |
| **EPICOR MEN** | ***P*_deltaOR_^*^** | **OR** | **95%CI** | ***P*** | **OR** | **95%CI** | | ***P*** | | | **OR** | **95%CI** | | ***P*** | |
| **RPMM-0[0.76] ^†^** |  | 1 | - | - | 1 | | - | | - | | 1 | | - | | - |
| **RPMM-1[0.72] ^†^** | 0.739 | 2.112 | 0.992-4.499 | 0.053 | 2.270 | | 0.886-5.813 | | 0.088 | | 2.635 | | 0.912-7.618- | | 0.074 |
| **RPMM-2[0.69] ^†^** | 0.948 | 3.783 | 1.672-8.560 | 0.001 | 3.726 | | 1.373-10.115 | | 0.010 | | 3.610 | | 1.138-11.446 | | 0.029 |
| **RPMM-3[0.65] ^†^** | 0.468 | 3.618 | 1.679-7.793 | 0.001 | 4.843 | | 1.833-12.769 | | 0.001 | | 5.956 | | 1.967-18.035 | | 0.002 |
| **LINE-1 methylation below the median** | 0.073 | 1.564 | 1.025-2.387 | 0.038 | 2.034 | | 1.215-3.405 | | 0.007 | | 3.214 | | 1.652-6.250 | | 0.001 |
|  |  |  |  |  |  | |  | |  | |  | |  | |  |
| **EPICOR WOMEN** | ***P*_deltaOR_^*^** | **OR** | **95%CI** | ***P*** | **OR** | | **95%CI** | | ***P*** | | **OR** | | **95%CI** | | ***P*** |
| **RPMM-0[0.75] ^†^** |  | 1 | - | - | 1 | | - | | - | | 1 | | - | | - |
| **RPMM-1[0.71] ^†^** | 0.395 | 2.346 | 0.863-6.377 | 0.095 | 3.973 | | 1.046-15.090 | | 0.043 | | 5.192 | | 1.118-24.123 | | 0.036 |
| **RPMM-2[0.68] ^†^** | 0.216 | 4.987 | 1.923-12.931 | 0.001 | 10.142 | | 2.743-37.507 | | 0.001 | | 15.640 | | 3.351-73.008 | | <0.001 |
| **RPMM-3[0.64] ^†^** | 0.440 | 6.250 | 1.991-19.619 | 0.002 | 14.974 | | 3.125-71.765 | | 0.001 | | 14.207 | | 2.484-81.260 | | 0.003 |
| **LINE-1 methylation below the median** | 0.284 | 1.170 | 0.653-2.095 | 0.598 | 1.725 | | 0.718-4.148 | | 0.223 | | 2.262 | | 0.787-6.502 | | 0.130 |
|  |  | Adj. by age and center of recruitment | | | MODEL 1 + smoke, BMI, physical activity, blood pressure, estimated WBC count, control probes PCs | | | | | | MODEL 2 + WHR, alcohol, LDL, HDL, triglycerides, economical index, glucose lowering medical treatments, menopausal status in women | | | | |

^*^see Suppl. Methods, *DNA methylation and MI risk* paragraph

^†^Mean Methylation value in square brackets

**Table S4A.** EPICOR case-control differential methylation analysis: comparison of models with and without lipids adjustments

| ***ZBTB12*-DMR**  **WITHOUT LIPIDS CORRECTION** | **effect-size** | **se** | **lower 95%CI** | **upper 95%CI** | ***P*_delta effect-size_** |
| --- | --- | --- | --- | --- | --- |
| **OVERALL** | -0.019 | 0.004 | -0.026 | -0.012 | - |
| **MEN** | -0.023 | 0.005 | -0.032 | -0.014 | - |
| **WOMEN** | -0.006 | 0.006 | -0.017 | 0.005 | - |
|  |  |  |  |  |  |
| ***ZBTB12*-DMR**  **WITH LIPIDS CORRECTION** | **effect-size** | **se** | **lower 95%CI** | **upper 95%CI** | ***P*_delta effect-size_** |
| **OVERALL** | -0.018 | 0.004 | -0.025 | -0.010 | 0.856 |
| **MEN** | -0.022 | 0.005 | -0.031 | -0.012 | 0.853 |
| **WOMEN** | -0.006 | 0.006 | -0.018 | 0.006 | 0.995 |
|  |  |  |  |  |  |
|  |  |  |  |  |  |
|  |  |  |  |  |  |
| **LINE-1**  **WITHOUT LIPIDS CORRECTION** | **effect-size** | **se** | **lower 95%CI** | **upper 95%CI** | ***P*_delta effect-size_** |
| **OVERALL** | -0.511 | 0.147 | -0.799 | -0.223 | - |
| **MEN** | -0.520 | 0.179 | -0.871 | -0.169 | - |
| **WOMEN** | -0.496 | 0.319 | -1.122 | 0.130 | - |
|  |  |  |  |  |  |
| **LINE-1**  **WITH LIPIDS CORRECTION** | **effect-size** | **se** | **lower 95%CI** | **upper 95%CI** | ***P*_delta effect-size_** |
| **OVERALL** | -0.679 | 0.165 | -1.003 | -0.356 | 0.447 |
| **MEN** | -0.891 | 0.219 | -1.320 | -0.461 | 0.191 |
| **WOMEN** | -0.496 | 0.339 | -1.161 | 0.169 | 0.999 |

**Table S4B.** EPIC-NL case-control differential methylation analysis: comparison of models with and without lipids, batch, and WBCs adjustments

| ***ZBTB12*-DMR**  **WITHOUT LIPIDS/WBCs/BATCH CORRECTION** | **effect-size** | **se** | **lower 95%CI** | **upper 95%CI** | ***P*_delta effect-size_** |
| --- | --- | --- | --- | --- | --- |
| **OVERALL** | -0.013 | 0.004 | -0.021 | -0.005 | - |
| **MEN** | -0.014 | 0.007 | -0.028 | -0.0003 | - |
| **WOMEN** | -0.012 | 0.004 | -0.020 | -0.004 | - |
|  |  |  |  |  |  |
| ***ZBTB12*-DMR**  **WITH LIPIDS CORRECTION** | **effect-size** | **se** | **lower 95%CI** | **upper 95%CI** | ***P*_delta effect-size_** |
| **OVERALL** | -0.017 | 0.004 | -0.024 | -0.009 | 0.510 |
| **MEN** | -0.013 | 0.008 | -0.028 | 0.003 | 0.894 |
| **WOMEN** | -0.010 | 0.005 | -0.020 | -0.0002 | 0.756 |
|  |  |  |  |  |  |
| ***ZBTB12*-DMR**  **WITH WBCs CORRECTION** | **effect-size** | **se** | **lower 95%CI** | **upper 95%CI** | ***P*_delta effect-size_** |
| **OVERALL** | -0.024 | 0.006 | -0.036 | -0.011 | 0.166 |
| **MEN** | -0.030 | 0.011 | -0.051 | -0.009 | 0.220 |
| **WOMEN** | -0.017 | 0.008 | -0.033 | -0.001 | 0.567 |
|  |  |  |  |  |  |
| ***ZBTB12*-DMR**  **WITH BATCH CORRECTION** | **effect-size** | **se** | **lower 95%CI** | **upper 95%CI** | ***P*_delta effect-size_** |
| **OVERALL** | -0.011 | 0.004 | -0.019 | -0.003 | 0.755 |
| **MEN** | -0.013 | 0.007 | -0.027 | 0.002 | 0.896 |
| **WOMEN** | -0.011 | 0.005 | -0.021 | -0.001 | 0.847 |
|  |  |  |  |  |  |
|  |  |  |  |  |  |
|  |  |  |  |  |  |
| **LINE-1**  **WITHOUT LIPIDS/WBCs/BATCH CORRECTION** | **effect-size** | **se** | **lower 95%CI** | **upper 95%CI** | ***P*_delta effect-size_** |
| **OVERALL** | -0.133 | 0.042 | -0.214 | -0.051 | - |
| **MEN** | -0.400 | 0.085 | -0.566 | -0.235 | - |
| **WOMEN** | -0.016 | 0.046 | -0.106 | 0.075 | - |
|  |  |  |  |  |  |
| **LINE-1**  **WITH LIPIDS CORRECTION** | **effect-size** | **se** | **lower 95%CI** | **upper 95%CI** | ***P*_delta effect-size_** |
| **OVERALL** | -0.124 | 0.043 | -0.208 | -0.040 | 0.885 |
| **MEN** | -0.373 | 0.086 | -0.542 | -0.204 | 0.822 |
| **WOMEN** | -0.014 | 0.048 | -0.108 | 0.081 | 0.975 |
|  |  |  |  |  |  |
| **LINE-1**  **WITH WBCs CORRECTION** | **effect-size** | **se** | **lower 95%CI** | **upper 95%CI** | ***P*_delta effect-size_** |
| **OVERALL** | -0.198 | 0.072 | -0.340 | -0.056 | 0.435 |
| **MEN** | -0.691 | 0.195 | -1.074 | -0.308 | 0.172 |
| **WOMEN** | -0.068 | 0.068 | -0.201 | 0.065 | 0.525 |
|  |  |  |  |  |  |
| **LINE-1**  **WITH BATCH CORRECTION** | **effect-size** | **se** | **lower 95%CI** | **upper 95%CI** | ***P*_delta effect-size_** |
| **OVERALL** | -0.106 | 0.043 | -0.190 | -0.023 | 0.662 |
| **MEN** | -0.349 | 0.085 | -0.516 | -0.182 | 0.669 |
| **WOMEN** | 0.014 | 0.050 | -0.084 | 0.111 | 0.667 |
