# Supplementary material for: Gene-specific DNA methylation profiles and LINE-1 hypomethylation are associated with myocardial infarction risk
Source: Clin Epigenetics. 2015 Dec 24;7:133. doi: 10.1186/s13148-015-0164-3 (PMC4690365; doi:10.1186/s13148-015-0164-3)
Supplement: Supplementary file 3 — Supplemental Figures S1, S2, S3, and S4. Figure S1. quantile-quantile plot, EPICOR overall subjects. Figure S2. quantile-quantile plot, EPICOR men. Figure S3. quantile-quantile plot, EPICOR women. Figure S4. locations of ZBTB12 and LINE-1 CpG sites investigated by Sequenom MassARRAY. CpGs (in red) investigated within ZBTB12-DMR, LINE-1, and flanking primers (upper case: complementary to DNA; lower case: T7-promoter sequence and 10mer tag). CpG sites that could not be tested individually due to MassARRAY technology constrains, but had to be tested jointly with neighboring CpGs as a single unit, are underlined: the methylation level is the cumulative value of all the sites within the CpG unit. (ZIP 91 kb) [file 13148_2015_164_MOESM3_ESM.zip › CLEP-D-15-00016-Suppl Figure S4 - MassArray CpGs locations.pdf]

ZBTB12

chr6:31867624-31868103, strand [+]

```
                                   32                               31    3029
CCCCAATTCTCCTGGGCCAAAGAGCCCTTTTTCCACGCAGCCCAGGGGCCCCAGCCTCCTGGCCTCCACGCCTGCGCGGC
   28        27       26                   25    24  23         22
TAGCGGATGAGGACGTTAATCTCGGCCACACTGGCCTCCAGCACGTTCTCGGCCGTGGTCTTGCCGTGTTGCTCCTTGAG
    21                    20   19  18 17            16       1514    13
GTGCCGCCTAATGGCAGGCTTGTGGGCGAAGCGCACGTCGCAGTAGGAGCAGCGGTAGGGCCGCGCTCCCGAGTGCAGGT
         12                               11       10  9                  8
TGAGGTGGTCGTGAAGGGTGGACTTCTGTGTGAAGCACTTGCCGCAGATGCCGCACGAGTGTGACTTGACACCACGATGC
 7          6                                      5                         4 .
ACGTTCATGTGGCGGTTGAGGTTGCTGCTGTGGTTGAACTGCTTGCCACAGCGAGGGCACATGAAGATGAAGTGCTGCGC
 3                        2               1
CCGCATGTGGAAGACCAGCTTCTCCACGCCCTGGAACACTTCCGGGCACTTGGTGCACTTGATGTTCTTTAAGGGGTTTC
```

LINE-1

Human LINE-1 transposon (L1Hs), GenBank: X58075.1

```
                                                   1
TGTGAGGTGTCAGTGTGCCCCTGCTGGGGGGTGCCTCCCAGTTAGGCTGCTCGGGGGTCAGGGGTCAGGGACCCACTTGA
              2                   3
GGAGGCAGTCTGCCCGTTCTCAGATCTCCAGCTGCGTGCTGGGAGAACCACTGCTCTCTTCAAAGCTGTCAGACAGGGAC

ACTTAAGTCTGCAGAGGTTACTGCTGTCTTTTTGTTTGTCTGTGCCCTGCCCCCAGAGGTGGAGCCTACAGAGGCAGGCA
                                  4        5
GGCCTCCTTGAGCTGTGGTGGGCTCCACCCAGTTCGAGCTTCCCGGCTGCTTTGTTTACCTAAGCAAGCCTGGGCAATGG
6   7                8     9                                        10      11 .
CGGGCGCCCCTCCCCCAGCCTCGTTGCCGCCTTGCAGTTTGATCTCAGACTGCTGTGCTAGCAATCAGCGAGATTCCGTG
  12
GGCGTAGGACCCTCTGAGCCAGGTGTGGGATA
```

Flanking Primers

```
CHR6_FOR:    aggaagagagGGGTTTTTTAGGTGGAAATTTTTTA
CHR6_REV:    cagtaatacgactcactatagggagaaggctCCCCAATTCTCCTAAACCAAAA
LINE-1_FOR:  aggaagagagGTGTGAGGTGTTAGTGTGTTTTGTT
LINE1_REV:   cagtaatacgactcactatagggagaaggctATATCCCACACCTAACTCAAAAAAT
```
