# Supplementary figures and images for: Gene-specific DNA methylation profiles and LINE-1 hypomethylation are associated with myocardial infarction risk
Source: Clin Epigenetics. 2015 Dec 24;7:133. doi: 10.1186/s13148-015-0164-3 (PMC4690365; doi:10.1186/s13148-015-0164-3)

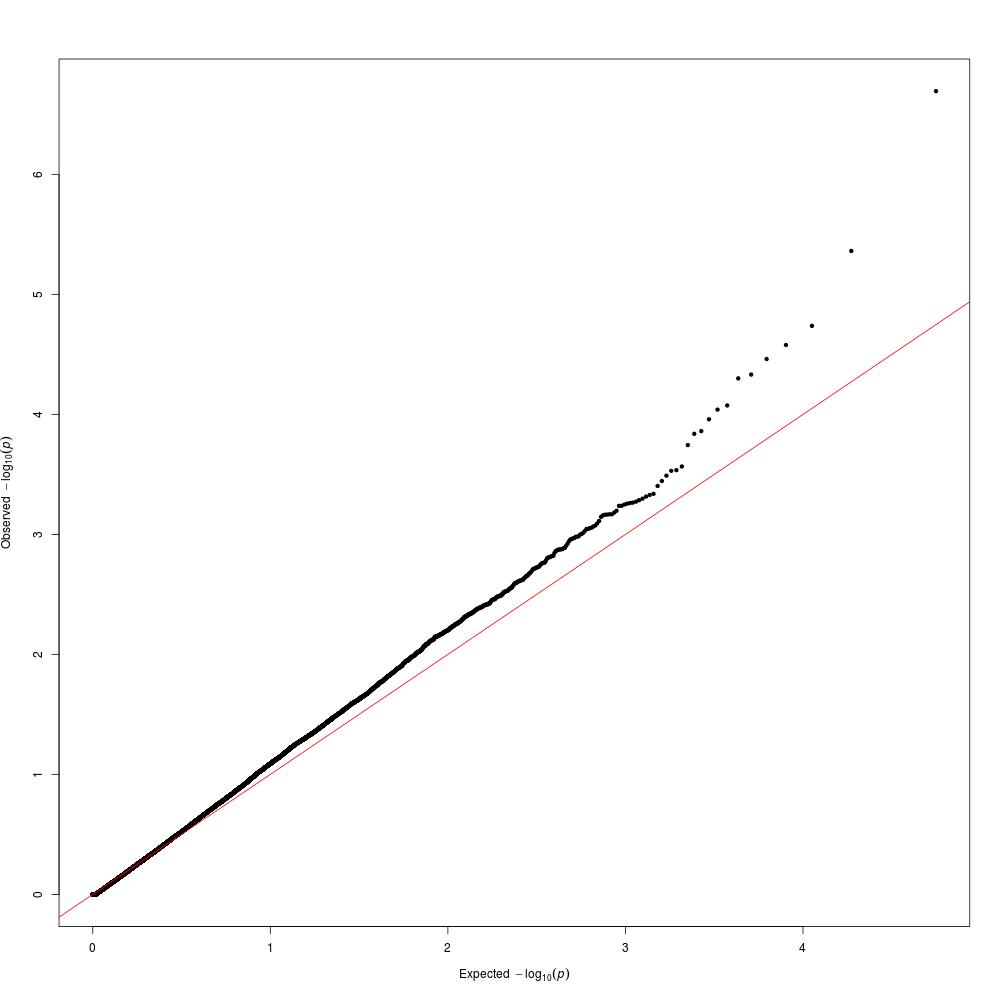

Supplement: Supplementary file 3 — Supplemental Figures S1, S2, S3, and S4. Figure S1. quantile-quantile plot, EPICOR overall subjects. Figure S2. quantile-quantile plot, EPICOR men. Figure S3. quantile-quantile plot, EPICOR women. Figure S4. locations of ZBTB12 and LINE-1 CpG sites investigated by Sequenom MassARRAY. CpGs (in red) investigated within ZBTB12-DMR, LINE-1, and flanking primers (upper case: complementary to DNA; lower case: T7-promoter sequence and 10mer tag). CpG sites that could not be tested individually due to MassARRAY technology constrains, but had to be tested jointly with neighboring CpGs as a single unit, are underlined: the methylation level is the cumulative value of all the sites within the CpG unit. (ZIP 91 kb) [file 13148_2015_164_MOESM3_ESM.zip › CLEP-D-15-00016-Suppl Figure S1 - QQplot_EPICOR overall.jpg]

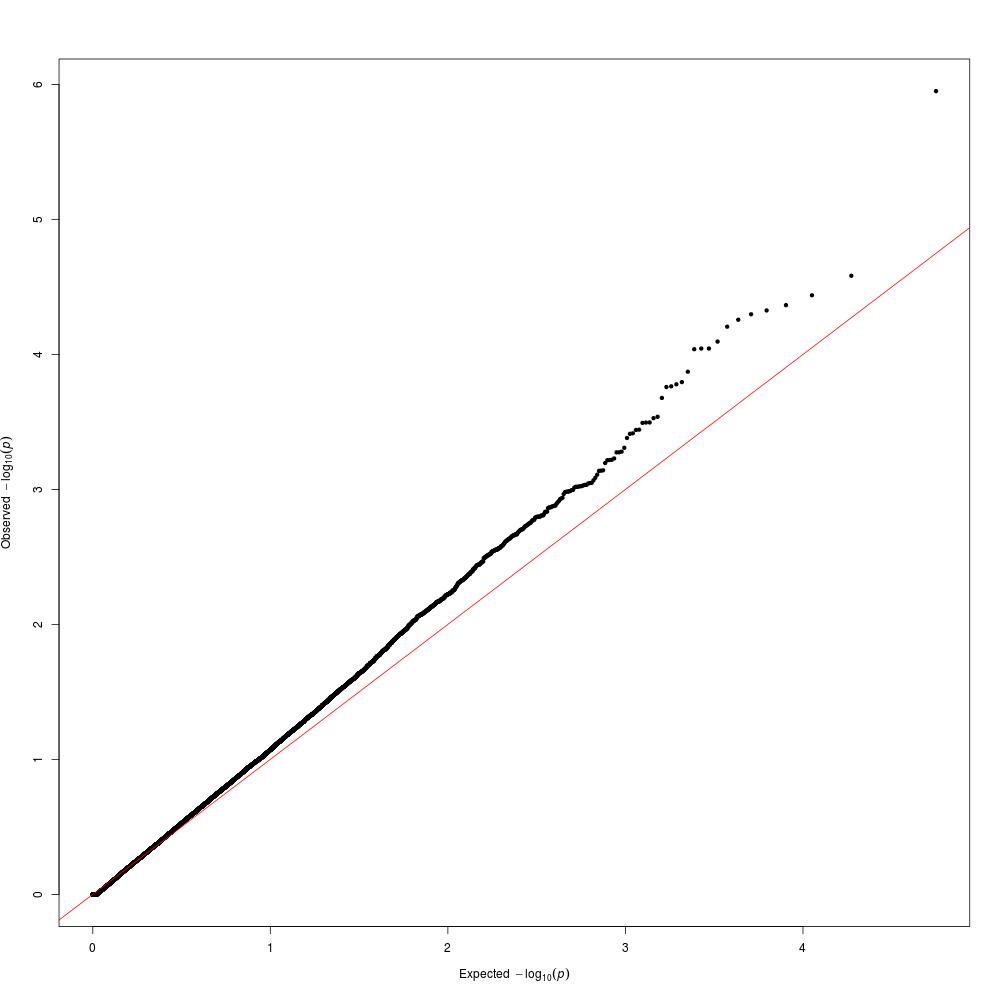

Supplement: Supplementary file 3 — Supplemental Figures S1, S2, S3, and S4. Figure S1. quantile-quantile plot, EPICOR overall subjects. Figure S2. quantile-quantile plot, EPICOR men. Figure S3. quantile-quantile plot, EPICOR women. Figure S4. locations of ZBTB12 and LINE-1 CpG sites investigated by Sequenom MassARRAY. CpGs (in red) investigated within ZBTB12-DMR, LINE-1, and flanking primers (upper case: complementary to DNA; lower case: T7-promoter sequence and 10mer tag). CpG sites that could not be tested individually due to MassARRAY technology constrains, but had to be tested jointly with neighboring CpGs as a single unit, are underlined: the methylation level is the cumulative value of all the sites within the CpG unit. (ZIP 91 kb) [file 13148_2015_164_MOESM3_ESM.zip › CLEP-D-15-00016-Suppl Figure S2 - QQplot_EPICOR men.jpg]

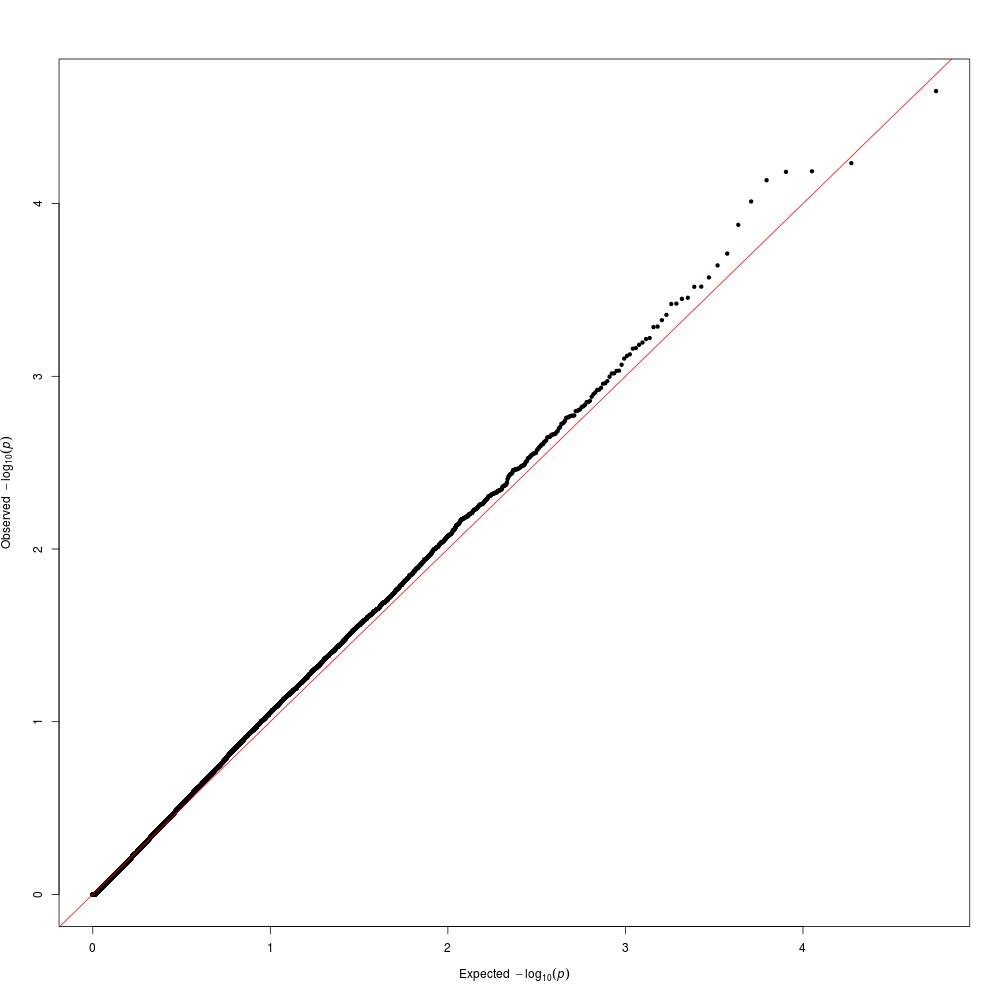

Supplement: Supplementary file 3 — Supplemental Figures S1, S2, S3, and S4. Figure S1. quantile-quantile plot, EPICOR overall subjects. Figure S2. quantile-quantile plot, EPICOR men. Figure S3. quantile-quantile plot, EPICOR women. Figure S4. locations of ZBTB12 and LINE-1 CpG sites investigated by Sequenom MassARRAY. CpGs (in red) investigated within ZBTB12-DMR, LINE-1, and flanking primers (upper case: complementary to DNA; lower case: T7-promoter sequence and 10mer tag). CpG sites that could not be tested individually due to MassARRAY technology constrains, but had to be tested jointly with neighboring CpGs as a single unit, are underlined: the methylation level is the cumulative value of all the sites within the CpG unit. (ZIP 91 kb) [file 13148_2015_164_MOESM3_ESM.zip › CLEP-D-15-00016-Suppl Figure S3 - QQplot_EPICOR women.jpg]
